# Quantifying the grapevine xylem embolism resistance spectrum to identify varieties and regions at risk in a future dry climate

**DOI:** 10.1038/s41598-023-34224-6

**Published:** 2023-05-12

**Authors:** Laurent J. Lamarque, Chloé E. L. Delmas, Guillaume Charrier, Régis Burlett, Ninon Dell’Acqua, Jérôme Pouzoulet, Gregory A. Gambetta, Sylvain Delzon

**Affiliations:** 1 Université de Bordeaux, INRAE, BIOGECO, 33615 Pessac, France; 2 Département des Sciences de l’Environnement, Université du Québec à Trois-Rivières, Trois-Rivières, QC Canada; 3 SAVE, INRAE, BSA, ISVV, 33882 Villenave d’Ornon, France; 4 Université Clermont Auvergne, INRAE, PIAF, 63000 Clermont-Ferrand, France; 5 EGFV, Bordeaux-Sciences Agro, INRAE, Université de Bordeaux, ISVV, 33882 Villenave d’Ornon, France

**Keywords:** Drought, Ecophysiology

## Abstract

Maintaining wine production under global warming partly relies on optimizing the choice of plant material for a given viticultural region and developing drought-resistant cultivars. However, progress in these directions is hampered by the lack of understanding of differences in drought resistance among *Vitis* genotypes. We investigated patterns of xylem embolism vulnerability within and among 30 *Vitis* species and sub-species (varieties) from different locations and climates, and assessed the risk of drought vulnerability in 329 viticultural regions worldwide. Within a variety, vulnerability to embolism decreased during summer. Among varieties, we have found wide variations in drought resistance of the vascular system in grapevines. This is particularly the case within *Vitis vinifera*, with varieties distributed across four clusters of embolism vulnerability. Ugni blanc and Chardonnay featured among the most vulnerable, while Pinot noir, Merlot and Cabernet Sauvignon ranked among the most resistant. Regions possibly at greater risk of being vulnerable to drought, such as Poitou–Charentes, France and Marlborough, New Zealand, do not necessarily have arid climates, but rather bear a significant proportion of vulnerable varieties. We demonstrate that grapevine varieties may not respond equally to warmer and drier conditions, and highlight that hydraulic traits are key to improve viticulture suitability under climate change.

## Introduction

Increasing temperatures and shifts in precipitation patterns pose serious threats to global crop production^1–3^, and maintaining high and consistent crop yields under drought and/or stricter water conservation policies is a major challenge for the sustainability of agricultural systems. Grapevine plays a significant role culturally and is the world’s third most valuable horticultural crop^4^. Wine growing regions worldwide have recently faced intense and frequent droughts and heat waves, e.g. 2009 in Australia, 2015 in California, and 2019 in France, and subsequent economical losses in wine production have been considerable^5^. Understanding how grapevine responds to extreme weather such as increasingly severe and sustained droughts is crucial to advise the wine industry about which varieties and viticultural practices would be best adapted to increased drought risk.

Adaptation of viticulture to climate change has first been approached from a phenological perspective^6–8^. Historically, specific grapevine varieties have been chosen for specific regions so that their phenological cycles match local climate^9^. For example, Pinot Noir and Riesling, which mature early, are grown in cooler regions, whereas late-ripening Grenache and Mourvedre are favored in hotter climates^10^. Phenological diversity within *Vitis vinifera* is high^8^, and it has been suggested that increasing diversity of varieties with different phenology may mitigate losses of agricultural areas and negative impacts of climate change^7^. However, plant productivity relies on water availability and photosynthetic capacity^11,12^ and therefore, optimizing phenology alone cannot increase drought tolerance in grapevine.

Regarding drought tolerance, grapevine stands out from major annual crops because of two features. First, it is a perennial crop that is expected to produce for many decades. Thus, it must tolerate drought periods over both short- and long terms, i.e. be able to produce annually while avoiding drought-induced mortality thresholds over the years^13^. Second, water deficit can improve berry and wine quality, especially for red wines, through increases in sugar, anthocyanin, and tannin concentration^14–16^. As a result, producers in regions where irrigation is allowed tend to restrict water to maximize production of high-quality grapes while minimizing yield reductions. For these reasons, a large body of work on grapevine physiology has focused on improving water use efficiency, notably attempting to elucidate the underlying mechanisms of stomatal regulation and photosynthesis limitation^17–21^. However, water use efficiency is often equated with drought resistance and improved crop yields under stress^22^, which is not the case^23^.

Indeed, water-carbon linkages in plants also rely on efficient hydraulic functioning that facilitate water movement from the soil up to the sites of photosynthesis. During prolonged drought, this system can be disrupted by air entry in water conducting xylem cells (c.f. xylem embolism)^12^, which become partially or entirely non-functional. Failure to maintain hydraulic conductivity impairs the photosynthetic yield and productivity of plants^24,25^, and ultimately limits survival under severe drought^26^. Yet, hydraulics in grapevine has been described in just a handful of common *Vitis vinifera* L. varieties, e.g. Cabernet Sauvignon^27,28^, Chardonnay^29^, Grenache^18,30^, Merlot^18,31^, and Syrah^18,30^. The magnitude of variation in vulnerability to xylem embolism within and among *Vitis* species and sub-species (varieties) is currently unknown, and limits our ability to provide growers and wine makers with robust recommendations for promoting specific varieties better adapted to warmer and drier climates. However, this knowledge is critical, whatever the viticultural management of water: whether this is in traditional vineyards of Europe where 90% of viticulture is rainfed^32^ and avoiding thresholds of hydraulic failure via irrigation is not an option in many regions, or in New World vineyards where irrigation might not prevent grapevines from reaching xylem embolism thresholds during midsummer under severe drought^18^.

In this study, we evaluated the global spectrum of vulnerability to xylem embolism in grapevine, with measurements conducted both within individual varieties over the growing season and between varieties originating from different locations and climates. We studied 30 grapevine varieties (Supplementary Table S1), encompassing (i) black-berried and white-berried *Vitis vinifera* varieties that collectively account for ~ 1,9 M hectares cultivated around the world and represent > 42% of the global winegrape bearing area, (ii) interspecific *Vitis* hybrid varieties, and (iii) commonly used *Vitis* rootstocks. Subsequently, these analyses allowed us to assess global wine regions with respect to their varietal diversity and resulting absolute risk of drought vulnerability. As a wide array of grapevine varieties are cultivated by humans, we hypothesized that the range of xylem vulnerability to embolism in grapevine would be relatively large, and thus, the risk of drought vulnerability would vary across vineyards worldwide.

## Results and discussion

### Variability in xylem embolism vulnerability over time within variety

**Figure 1:**
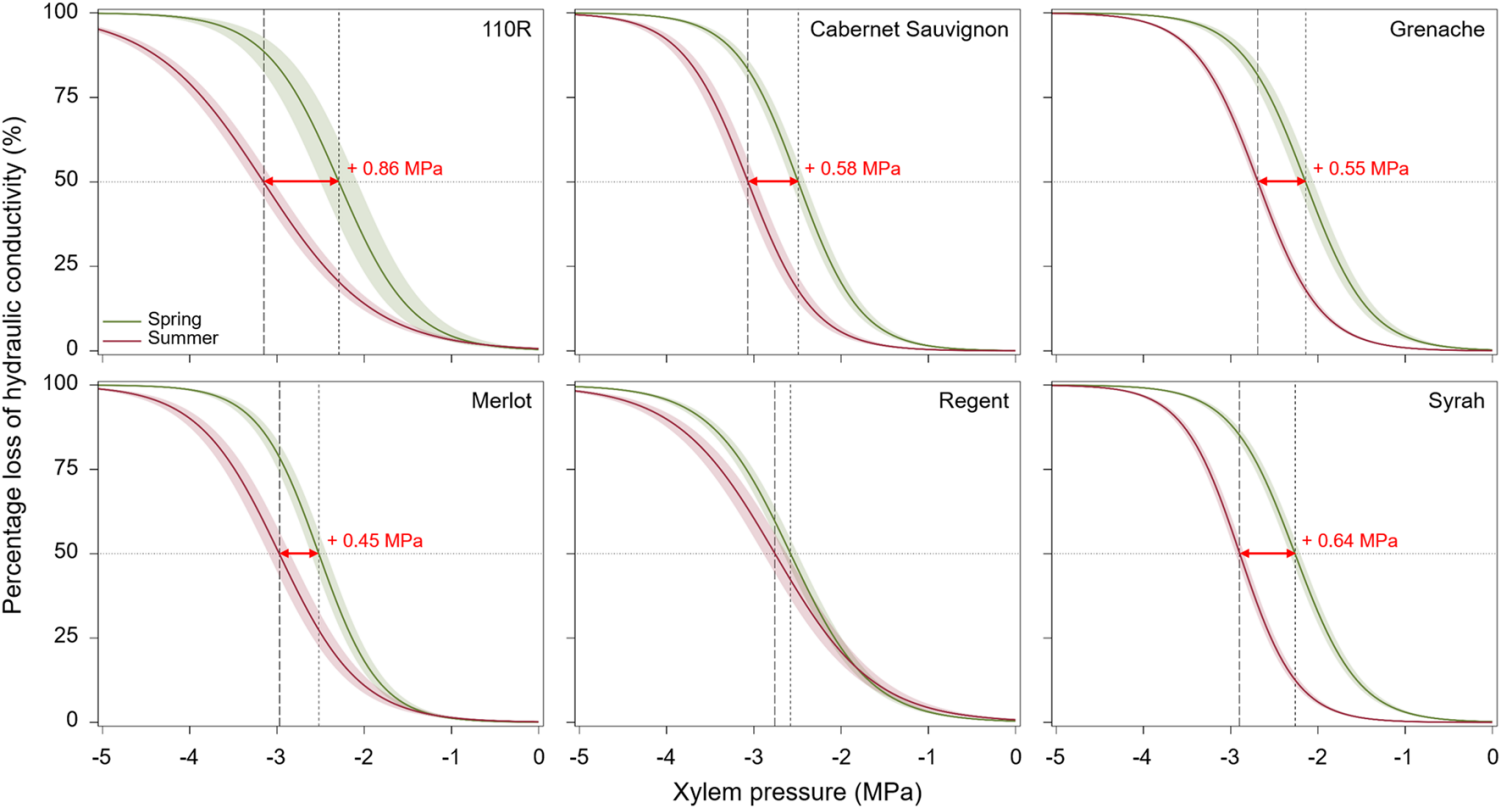
Variability in stem vulnerability to xylem embolism over the course of the growing season. Mean spring and summer vulnerability curves (VCs), expressed as percentage loss of hydraulic conductivity (PLC, %), in four *Vitis vinifera* varieties (Cabernet Sauvignon, Grenache, Merlot, and Syrah), one hybrid variety (Regent), and one rootstock (110R). Spring and summer VCs are colored in green and red, respectively. Shaded bands represent standard errors. Horizontal dotted lines indicate the 50% embolism threshold, while red double-headed arrows and red values depict the change in *Ψ*_50_ over the course of the growing season (i.e. ontogenic effect). Measurements were conducted on one-year-old plants. See Supplementary Table S10 for sample sizes, means ± SE and results of statistical tests.

Previous surveys of water potentials in vineyards showed that the severity of drought stress increases throughout the growing season^18,33^. Thus, if some hydraulic adjustment occurs in grapevine to cope more easily with this situation, we would expect a seasonal decrease in vulnerability to xylem embolism. To examine this, we first compared vulnerability to xylem embolism in stems between spring and summer in four *Vitis vinifera* varieties, one hybrid variety, and one rootstock, using the in situ flow-centrifugation technique equipped with a 100-cm diameter rotor^34^. We found that stem vulnerability to xylem embolism decreased significantly along the season in all varieties but Regent (i.e. ontogenic effect). The difference in xylem pressure inducing 50% embolism (c.f. *Ψ*_50_) between spring and summer ranged from 0.45 MPa in Merlot to 0.88 MPa in 110R (Fig. 1, Supplementary Table S2).

The temporal decrease in stem vulnerability to xylem embolism was further observed from year to year, as the xylem pressure inducing 50% and 88% embolism (c.f. *Ψ*_88_) in spring was 0.36 MPa and 0.56 MPa more negative in the current-year shoot of two-year-old Syrah plants, compared to their one-year-old counterparts (Supplementary Table S3).

We next tested whether seasonal changes in vulnerability to xylem embolism also occurred in mature leaves, using the non-invasive, optical technique^35^ on intact plants. First, measurements in Cabernet Sauvignon showed that spring and summer leaves were equally resistant to xylem embolism (Supplementary Table S4, Supplementary Fig. S1). However, there appeared to be a trend toward lower embolism thresholds as the season progressed. Regardless of leaf nodal positions, 50% embolism was reached at water potentials around − 1.8 MPa in spring and − 2.0 MPa in summer, whereas 88% embolism threshold averaged − 2.2 MPa in spring and − 2.4 MPa in summer. In addition, observations in the field and during experimental dry-downs^29,30^ have indicated that, when grapevine is exposed to severe drought, leaf shedding primarily occurs in basal leaves formed earlier in the season compared to apical leaves. Thus, examination of leaf embolism vulnerability variation throughout the season was further conducted at two different nodal positions along the stems. Given that basal leaves experience less negative water potentials than apical leaves, we hypothesized that the discrepancy in the timing of leaf shedding would result from a difference in embolism vulnerability, with basal leaves being more vulnerable to embolism. Measurements in Cabernet Sauvignon and Syrah plants showed that basal leaves constantly exhibited higher embolism vulnerability compared to apical leaves (Supplementary Table S4, Supplementary Fig. S1). In particular, water potential inducing 88% embolism was 0.3–0.4 MPa higher in basal than apical leaves in Cabernet Sauvignon in spring and summer, and 0.6 MPa higher in basal than apical leaves in Syrah in spring. Overall, the range of leaf *Ψ*_50_ values obtained in this study, going from − 1.7 MPa to − 2.2 MPa in Cabernet Sauvignon and from − 1.5 MPa to − 1.8 MPa in Syrah, was in line with the 50% embolism thresholds reported previously in leaves in these varieties^28,30^.

The seasonal dynamics of xylem embolism vulnerability observed in stems, and to a lesser extent in leaves, emphasize the ability of grapevine to rapidly adapt its hydraulic system to increasingly drier conditions along the summer. These results support previous findings in two ways. First, they match those that have documented a decrease in leaf embolism vulnerability in *Vitis vinifera* cv. Tempranillo, Grenache and Cabernet Sauvignon as summer progressed^28,33^. Similar changes in xylem embolism vulnerability along the course of plant development have also been described in other crops^36^ and woody species^37,38^. Second, they concur with studies that found substantial plasticity in xylem embolism vulnerability of various species in response to experimentally-induced variation in water^24,39^, light^40^, and nutrient^41^ availability. Moreover, there is strong evidence that, in grapevine, leaf physiological traits such as stomatal closure and turgor loss point also display a high degree of plasticity along the growing season^28,31,33,42^. Thus, the seasonal shift in xylem embolism vulnerability is coordinated with acclimation of other traits to increased water deficit^28,33^. Hydraulic and physiological traits are part of an integrated strategy aiming at maintaining a positive safety margin, that is the difference between midday water potentials (*Ψ*_min_) and the onset of embolism, in order to protect the hydraulic system when water potentials decrease throughout summer. The fact that the seasonal decrease in stem embolism vulnerability was observed in plants that were maintained in a glasshouse under well-watered conditions before measurements further suggests that this is a pre-determined strategy that takes place independently from the seasonal increase in drought conditions, possibly in relation to increased lignification and changes in wood anatomical features through time^43,44^.

Regent was the only variety in which the decrease in stem embolism vulnerability along the season was not statistically significant. The difference in seasonal plasticity between Regent and the other grapevines, in particular the four *Vitis vinifera* varieties, might be explained by the fact that Regent is a *Vitis* interspecific hybrid originating from the crossing of Diana (Sylvaner x Müller-Thurgau) with Chambourcin, the latter being also a French-American interspecific hybrid. It is thus possible that the plastic response of vulnerability to xylem embolism that is observed in *Vitis vinifera* has been lost during the breeding processes with American wild grapevines (e.g. *Vitis berlandieri*, *V*. *rupestris*, and *V*. *riparia*). However, further investigation to this effect is needed, especially since the rootstock 110R, with a *V*. *berlandieri* x *V*. *rupestris* genetic background, exhibited significant plasticity in stem embolism vulnerability along the season, and American grapevines such as *V*. *candicans*, *V*. *labrusca* and *V*. *rupestris* were found equally resistant to xylem embolism than *V*. *vinifera* varieties^45^.

Finally, measurements of xylem embolism vulnerability in stems and leaves in Cabernet Sauvignon and Syrah allowed us to assess the hypothesis of hydraulic vulnerability segmentation. This hypothesis posits that distal organs (e.g. leaves) are more vulnerable to xylem embolism than perennial organs (e.g. stems) in order for plants to protect permanent, high-investment tissues^46^. Comparisons between organs showed that across the two varieties, leaves exhibited significant higher vulnerability to embolism than stems. For instance, values of *Ψ*_50_ in leaves were reached at water potentials 21% to 37% higher compared to stems. This led to positive hydraulic vulnerability segmentation (HVS), regardless of the season and leaf position along the stems (Supplementary Table S4). The HVS_*Ψ*12_ ranged from 0.19 MPa in Syrah in spring to 0.47 MPa in Cabernet Sauvignon in summer, and HVS_*Ψ*50_ from 0.62 MPa to 1.02 MPa (Supplementary Fig. S2). As *Ψ*_50_ was generally lower for apical than basal leaves, the resulting hydraulic vulnerability segmentation tended to be smaller at the top than at the base of stems (Supplementary Table S4, Supplementary Fig. S2). These results are consistent with previous studies that documented through micro-computed tomography higher embolism vulnerability in grapevine leaf petioles compared to stems^27,47^. The hydraulic vulnerability segmentation appears to be a widespread strategy among grapevine varieties to prevent perennial organs from undergoing severe hydraulic dysfunction under severe drought.

### Variability in xylem embolism vulnerability among species and varieties

The evaluation of drought-induced xylem embolism along the season proved that controlling for the seasonal timing of measurements was a prerequisite for examining the range of variation in xylem embolism vulnerability among grapevine species and varieties. Thus, we studied the global spectrum of grapevine embolism vulnerability by conducting in situ flow-centrifugation measurements in summer exclusively. We aimed here at improving the mechanistic understanding of differences in drought tolerance in *Vitis*. Xylem embolism vulnerability in stems was compared across 220 plants from 30 *Vitis* genotypes, including 22 *Vitis vinifera* varieties of different subspecies (proles) and geographical origins, five hybrid varieties, and three rootstocks. In the current context of viticulture adaptation to climate change, the objective was twofold: expanding our ability to inform of the optimum choice of plant material for a given viticultural landscape, and enhancing selection of key traits linked to grapevine yield maintenance and survival under drought stress that would be of high interest in phenotyping and breeding approaches. In addition, studies have observed inter-varietal differences in stomatal closure and transpiration rates when grapevines are subjected to drought^30,33^. If the coordination between embolism vulnerability and physiological traits that was observed over the season for a given variety also holds true across genotypes, we would expect significant differences in embolism vulnerability among grapevine varieties grown under the same conditions (‘common garden’^48^).

**Figure 2:**
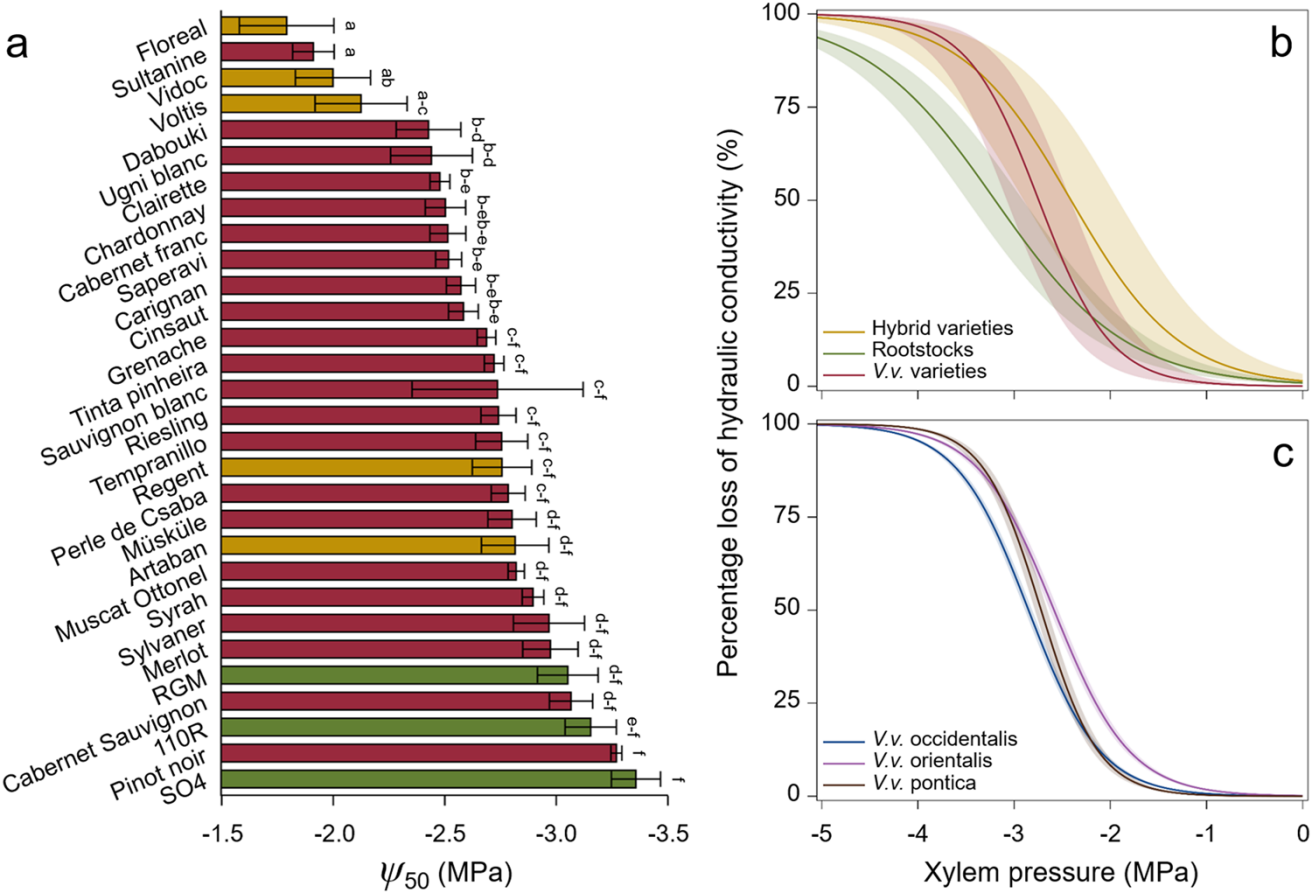
Variability in stem vulnerability to xylem embolism among grapevine varieties. (**a**) Range of *Ψ*_50_ (%) for the 30 grapevine varieties screened. Bars and errors represent means ± standard errors. Letters refer to the results of post-hoc tests. Different letters indicate significant differences among varieties. See Supplementary Table S1 for means ± SE. (**b**) Mean vulnerability curves (VCs), expressed as percentage loss of hydraulic conductivity (PLC, %), of grapevine variety types. *n* = 22, 5, and 3 for *Vitis vinifera* (*V.v.*) varieties, interspecific hybrid varieties, and *Vitis* rootstocks, respectively. Shaded bands represent standard deviations. (**c**) Mean vulnerability curves (VCs) for *Vitis vinifera* (*V.v.*) subspecies. *n* = 12, 7, and 3 for *Vitis vinifera* subsp. (proles) occidentalis, orientalis, and pontica, respectively. Shaded bands represent standard errors. All measurements were conducted in summer.

We found that stem vulnerability to xylem embolism in summer varied significantly among the 30 grapevine varieties screened. Xylem pressure inducing 12%, 50% and 88% embolism ranged from − 0.4 to − 2.7 MPa, − 1.8 to − 3.4 MPa, and − 2.9 to − 5.0 MPa, respectively (Fig. 2a, Supplementary Fig. S3; see also Supplementary Table S5 for means per variety). This pattern was notably driven by a significant difference among the types of varieties (interspecific hybrids vs. rootstocks vs. *Vitis vinifera* varieties). Hybrid varieties showed the highest mean vulnerability to xylem embolism as Floreal, Vidoc and Voltis ranked among the four most vulnerable varieties (Fig. 2a). In contrast, the onset of embolism (c.f. *Ψ*_12_) in rootstocks and *Vitis vinifera* varieties was reached at a xylem pressure being almost twice more negative (Fig. 2b, Supplementary Table S6). Rootstocks further displayed a significant 17% to 33% increase in *Ψ*_50_ (i.e. more negative values) and a 30% increase in *Ψ*_88_ compared to *Vitis vinifera* and hybrid varieties (Supplementary Table S6).

**Figure 3:**
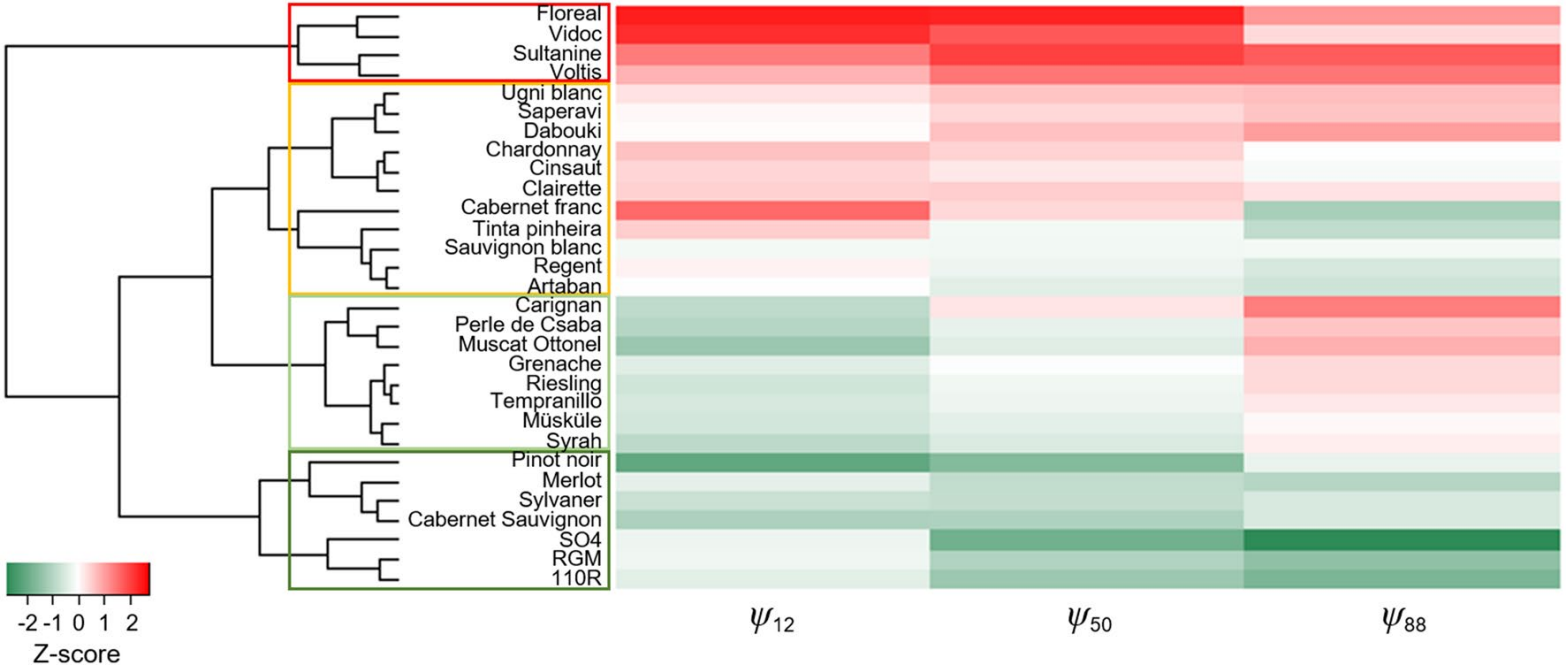
Hierarchical clustering analysis of the 30 grapevine varieties screened based on the varietal *Ψ*_12_, *Ψ*_50_ and *Ψ*_88_ values. The heatmap color scale represents Z-score normalized trait values (Z-score = (observed value–mean value)/standard deviation), going from red for high (less negative) values of *Ψ*_12_/*Ψ*_50_/*Ψ*_88_ to green for low (more negative) values. *Vitis* varieties were organized into four clusters of vulnerability to xylem embolism: low (dark green rectangle), low-to-medium (light green rectangle), medium-to-high (orange rectangle), high (red rectangle).

The variation in embolism resistance among varieties was further observed with a hierarchical clustering analysis carried out from the *Ψ*_12_/*Ψ*_50_/*Ψ*_88_ values and that distinguished four classes of vulnerability to xylem embolism (Fig. 3). The three rootstocks (110R, RGM and SO4) featured among the low embolism vulnerability class, whereas three of the five hybrid varieties (Floreal, Vidoc and Voltis) belonged to the high embolism vulnerability class. The slope (*S*) of the vulnerability curves differed both among the 30 varieties (Supplementary Fig. S3) and among the types of varieties (Fig. 2b). It was the steepest in *Vitis vinifera* varieties (Supplementary Table S6), which meant that embolism propagation through the xylem network is faster than in hybrid varieties and rootstocks. There was no difference in *S* values between hybrid varieties and rootstocks.

**Figure 4:**
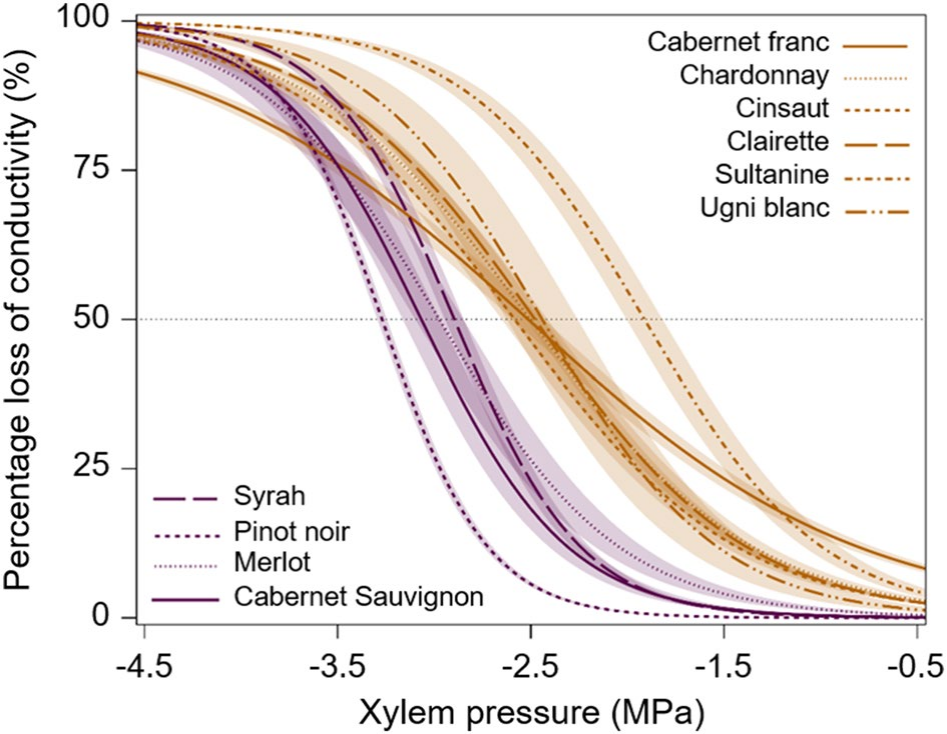
Mean vulnerability curves (VCs) of the most vulnerable and resistant *Vitis vinifera* varieties regarding summer *Ψ*_12_ and *Ψ*_50_. VCs are expressed as percentage loss of hydraulic conductivity (PLC, %). The vulnerable and resistant varieties are colored in orange and violet, respectively. Shaded bands represent standard errors. Horizontal dotted lines indicate the 50% embolism threshold. All measurements were conducted in summer.

We next examined whether embolism vulnerability varied specifically within *Vitis vinifera*. Analyses highlighted significant intraspecific variability, with varieties distributed along a range of *Ψ*_12_ of 2 MPa and a range of 1.3 MPa for both *Ψ*_50_ and *Ψ*_88_ (Fig. 2a, Supplementary Fig. S3). This pattern was partly driven by Sultanine (a.k.a. Thompson Seedless), which exhibited the least negative xylem pressure inducing 12%, 50% and 88% embolism (Supplementary Table S5). For instance, Sultanine had a *Ψ*_12_ three times higher and a *Ψ*_50_ ~ 40% higher than Pinot noir (*Ψ*_12_ = − 0.9 vs. − 2.7 MPa and* Ψ*_50_ = − 1.9 vs. − 3.3 MPa). Other vulnerable varieties with less negative *Ψ*_12_ and *Ψ*_50_ included Cabernet franc, Chardonnay, Cinsaut, Clairette, and Ugni blanc (Figs. 3 and 4). On the contrary, Cabernet Sauvignon, Merlot, Pinot noir, and Syrah displayed high resistance to xylem embolism by constantly showing significantly lower *Ψ*_12_ and *Ψ*_50_.

Differences in vulnerability to xylem embolism within *Vitis vinifera* were also observed with respect to subspecies (proles), geographic origin and berry skin colors. Thresholds of 12%, 50% and 88% embolism across *Vitis vinifera* subsp. (proles) occidentalis, orientalis, and pontica were distributed along a range of 0.2–0.4 MPa (Supplementary Table S7). Proles orientalis significantly exhibited less negative *Ψ*_12_ and *Ψ*_50_, while proles occidentalis showed the lowest *Ψ*_88_ (Fig. 2c). Regarding the geographical origins of varieties, there was no difference in *Ψ*_12_ among the four geographical groups (Supplementary Table S8). Yet, varieties from the Iberian Peninsula and from Western and Central Europe significantly exhibited *Ψ*_50_ and *Ψ*_88_ values that were 0.4–0.5 MPa lower compared to varieties from Balkans. The *Ψ*_88_ of varieties from the Iberian Peninsula was also significantly lower than that of varieties from the Eastern Mediterranean and Caucasus region. Finally, even though white- and black-berried varieties did not differ in *Ψ*_12_, white-berried varieties were slightly more vulnerable to xylem embolism than black-berried varieties with respect to *Ψ*_50_ and *Ψ*_88_ (Supplementary Table S9). Thresholds of 50% and 88% embolism in white-berried varieties were significantly reached at a xylem pressure 0.2 MPa higher than in black-berried varieties.

The examination of the embolism vulnerability spectrum across grapevine varieties drew several observations that contribute to viticulture adaptation under warmer and drier climate. It is important to note that vulnerability to embolism of a given variety appears to be similar regardless of the type of material measured, i.e. rooted cuttings or grafted scions^18^. First, our results highlighted that grapevine can sustain significant levels of water deficit before embolism occurs (mean *Ψ*_12_ = − 1.8 MPa in *Vitis vinifera*). This is in accordance with recent imaging technique-based studies^27,29,30,49^ reporting that grapevine is more resistant to xylem embolism than initially suggested (*Ψ*_50_ > − 1 MPa)^50,51^. This strategy is meaningful in two ways: many vineyards worldwide are located in arid and semi-arid regions characterized by frequent drought stress^52^, and even irrigated during the growing season, the hydraulic system of grapevine remains under tension^18,29,53^, which makes embolism repair highly unlikely^27^. Second, comparing our dataset to midday water potentials (*Ψ*_min_) monitored in vineyards during rain-fed experiments indicated that grapevine varieties barely reach water potentials associated with critical thresholds of xylem embolism in perennial organs. This is particularly the case for the commonly studied *Vitis vinifera* cv. Cabernet Sauvignon, for which reported *Ψ*_min_ in vineyards were not lower than − 1.6 MPa^28,53,54^ whereas the onset of embolism (c.f. *Ψ*_12_) in stem was found at − 2.2 MPa. Other instances include *Vitis vinifera* cv. Merlot, whose stem *Ψ*_12_ of − 1.8 MPa is lower than *Ψ*_min_ of − 1.5 MPa measured in Israel across the 2011–2012 seasons^55^, and *Vitis vinifera* cv. Tempranillo, whose stem *Ψ*_12_ = − 1.9 MPa is lower than *Ψ*_min_ of − 1.4 MPa monitored in Spain across the 2000–2004 seasons^56^. This result supports previous observations that grapevine tends to operate within a safe margin of water potentials where embolism is rare^18^. This is possible because during drought grapevine continually adapts its functioning to avoid hydraulic failure, notably through stomatal closure limiting water loss^28,30^. In other words, grapevine aims at securing continuous water supply even if it is at the expense of carbon assimilation, and therefore, grape yield and quality. However, the scarcity of multi-year *Ψ*_min_ records under field conditions for the majority of varieties makes extrapolating this strategy to the whole *Vitis vinifera* taxa difficult. Such information would notably be required for varieties such as Chardonnay and Sauvignon Blanc, which show higher embolism vulnerability than Cabernet Sauvignon and Merlot while being abundantly cultivated in regions like Australia and South Africa where fresh water resources for agriculture are scarce^57^.

Third, varieties of *Vitis vinifera* were found to vary in *Ψ*_12_ and *Ψ*_50_ by 2.0 MPa and 1.3 MPa, respectively, with corresponding intraspecific coefficients of variation (CV_intra_) of 25.8% and 10.3%. *Vitis vinifera* exhibited high intraspecific variability in embolism vulnerability compared to what has generally been reported in other species, provided that provenances or populations were similarly grown in a common environment. For example, CV_intra_ of stem *Ψ*_50_ ranged between 0.7% in *Pinus pinaster*^58^ to 4.12% in *Fagus sylvatica*^59^ to 7.6% in *Quercus douglasii*^60^, whereas variation in *Ψ*_50_ among cultivars of *Helianthus annuus* and *Juglans regia* was < 0.2 MPa^61,62^. This result is particularly striking considering the history of grapevine domestication and the combined action of migration, selection and admixture in shaping *Vitis vinifera* genetic structure^63^. Humans have selected traits improving productivity, berry size, sugar and acidity content^64^, and it remains unclear to what extent they have, purposely or not, selected for hydraulic traits related to drought resistance. Alternatively, significant variation arose both within and among subspecies (proles) and geographical groups. It is thus possible that divergent selection might have acted as another force enhancing genetic differentiation in embolism vulnerability among varieties.

We also found that interspecific hybrid varieties that are resistant to mildews were significantly more vulnerable to xylem embolism than *Vitis vinifera* varieties. This is notably the case of Floreal, Vidoc, and Voltis, which together showed a *Ψ*_12_ (− 0.68 MPa) three times less negative and a *Ψ*_50_ (− 1.97 MPa) reduced by a third compared to the mean *Ψ*_12_ and *Ψ*_50_ of *Vitis vinifera*. This result is worrisome for two reasons. In light of long-term monitoring of water potentials in vineyards showing that *Ψ*_min_ commonly falls below − 1 MPa during the growing season^18^, our finding suggests that hybrid varieties would be prone to substantial hydraulic failure on a regular basis throughout summer. Moreover, considering that in grapevine, like in plants in general, stomata close before the onset of embolism to operate within a positive hydraulic safety margin^28,30^, high vulnerability of these hybrid varieties suggests that they may also have more limiting stomatal regulation, photosynthetic capacities, and therefore productivity, under drought. Clearly, further investigations regarding their physiological functioning are required. Overall, the higher embolism vulnerability of hybrid varieties represents striking evidence that breeding approaches dedicated to the resistance to pathogens and/or yield maintenance under abiotic stress must actively account for hydraulic traits associated with resistance to hydraulic failure under drought conditions.

Finally, hydraulic properties of water conducting cells strongly rely on wood anatomical features^12,45,65^. Thus, we tested whether differences in xylem embolism vulnerability among grapevine varieties were related to discrepancy in xylem anatomy, and carried out observations from stem pieces collected in 19 *Vitis vinifera* and hybrid varieties during centrifugation measurement campaigns. Grapevine varieties showed similar xylem anatomical features, as illustrated by comparable vessel density (Supplementary Table S10, Supplementary Fig. S4a). There was no major difference either between types of varieties, with *Vitis vinifera* varieties having similar mean xylem area, vessel diameter, and vessel density than hybrid varieties (Supplementary Table S11, Supplementary Figs. S4a and S5). Across varieties, vessel density was negatively and significantly correlated to weighted hydraulic diameter (*df* = 1, 30, *F* = − 4.45, *P* = 0.0001), with a very low density of vessels wider than 100 µm (Supplementary Fig. S4). There was no significant correlation between hydraulic and xylem anatomical traits (Supplementary Table S12, Supplementary Fig. S4b). Only *Ψ*_50_ and *Ψ*_88_ correlated marginally with theoretical specific hydraulic conductivity (*k*_th_), as varieties that exhibited lower vulnerability to xylem embolism showed lower *k*_th_. Overall, our results revealed that grapevine varieties differed little with respect to the xylem anatomical traits examined. In the literature, differences have been observed between some varieties^66–68^, but histological data suggest that variation in xylem anatomy likely depends on which varieties and anatomical traits are evaluated^68^. The lack of correlation between xylem embolism vulnerability and xylem anatomical traits measured further supports observations that the vessel size-embolism vulnerability trade-off is complex and involves multiple anatomical, mechanical, and chemical xylem characteristics^69–71^. It remains to be understood how these features are integrated within a single strategy of drought resistance in grapevine.

### Regional risk index of drought vulnerability

Climate change is expected to intensify drought periods worldwide, which poses a serious threat to global wine production as the vast majority of traditional wine growing regions are already located in temperate and Mediterranean zones with warm and dry summers. However, in line with the scarcity of data on xylem embolism vulnerability in grapevine, risks of drought vulnerability (in an absolute sense) at higher spatial resolution across wine growing regions have yet to be characterized. To examine this, the varietal embolism vulnerability dataset was coupled with a database of regional and national winegrape bearing areas by variety^72^ (Supplementary Data S1, Supplementary Fig. S6a,b for examples on France and New Zealand). Here, the regional risk of drought vulnerability was voluntarily examined under a no-irrigation (dry-farmed) scenario, because (i) the majority of vineyards in Europe are rainfed, and (i) irrigation, that becomes increasingly necessary in hotter regions worldwide, will simply increases competition for fresh-water resources, making the search for varieties better adapted to drier climate all the more relevant. We calculated the bearing area of each embolism vulnerability cluster per wine region and country (Supplementary Fig. S6c,d for examples on France and New Zealand), and estimated a risk index of large-scale drought vulnerability for each region for which the varietal coverage of our dataset represented at least 40% of the regional or national winegrape bearing area (Supplementary Data S2). Thus, the regional risk index of drought vulnerability was reported for a total of 329 wine producing regions spanning 37 countries around the world, which together represented 52% of the global wine producing regions.

**Figure 5:**
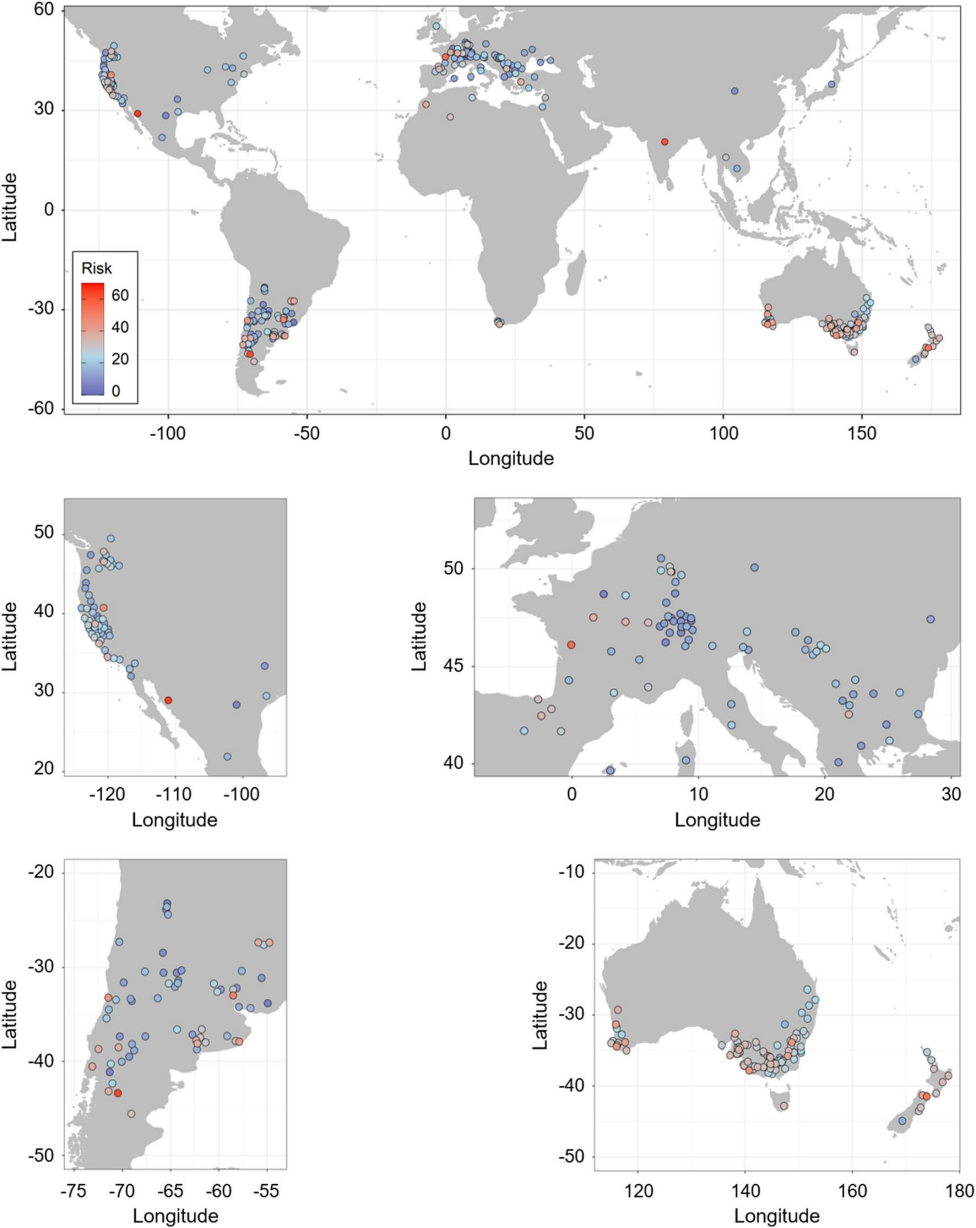
Regional risk index of drought vulnerability. The index is presented for regions and countries where *Vitis* varieties included in this study cumulatively cover  ≥ 40% of the winegrape bearing area (see also Supplementary Data S2).

The risk index varied between 5 and 65 but ranged between 20 and 40 for 56% of the regions (Fig. 5). The regions with the lowest risk index, such as Lavalleja (Uruguay), Humahuaca (Argentina), Ile de France (France) and Uri (Switzerland), corresponded to areas that were almost exclusively covered by the most embolism resistant varieties (c.f. Cabernet Sauvignon, Merlot and Pinot noir). On the contrary, Marlborough (New Zealand), Poitou–Charentes (France), Languiñeo (Argentina) and Sonora (Mexico) displayed the highest risk of drought vulnerability as they mostly grow varieties with the greatest vulnerability to embolism. Indeed, Sultanine monopolizes 72% of the bearing area in Sonora, Sauvignon blanc 78% in Marlborough, Ugni blanc 85% in Poitou–Charentes and Chardonnay 100% in Languiñeo. India globally also showed a high index as ~ 40% of the country bearing area is covered by Sultanine.

More generally, two main observations stood out from these analyses. First, the risk of drought vulnerability at the regional scale varies independently from the number of grapevine varieties grown (Pearson’s *r*^*2*^ < 0.1, *P* > 0.5). In other words, regions that are at lower risk of large-scale drought vulnerability are not necessarily the ones bearing the highest number of varieties. This illustrates that from a physiological point of view, embolism vulnerability of specific varieties matters more than the diversity of varieties grown regionally. This result softens conclusions of recent studies that have advocated for the use of phenological diversity to mitigate the negative effects of climate change on wine production^7,8^. A thorough evaluation of possible avenues to adapt viticulture to warmer and drier conditions should certainly account for inter-varietal variation in hydraulic traits related to drought resistance^45^. This would particularly be necessary in the context of northward expansion of wine growing regions like in England, where the choice of materials has relied on matching variety phenology with local climate^8^. Second, the regional risk index of drought vulnerability varies independently from climate and shows a spatial heterogeneity around the world. Regions that have a greater risk index of drought vulnerability are not necessarily those from arid and semi-arid zones. For instance, Rheingau in Germany, Spanish Basque Country and Peel in Australia exhibit similar risk indexes even though they are located in continental, oceanic, and Mediterranean zones, respectively. In other words, growers have produced wine according to variety characteristics irrespective of xylem embolism resistance (e.g. timing of ripeness). This result indicates that no traditional wine growing region is immune to impacts of climate change regarding the risk of xylem embolism during prolonged drought. Yet, although the majority of grapevine bearing areas have experienced substantial warming during the last decades^73^, patterns of increasing temperatures are variable across latitudes and seasons^74^. In consequence, since ultimately hydraulic failure results from the relationship between the minimum water potentials measured in the field and the innate vulnerability of the varieties grown there, to what extent regions differ in vulnerability to drought in a more general sense remains an open question.

## Conclusion

Grapevine shows a strong capacity to rapidly adjust to increasing drought stress during summer by strengthening the resistance of water transport pathway to hydraulic failure. Intraspecific comparisons further highlighted that *Vitis vinifera* varieties display significant variability in the vulnerability to xylem embolism. As grapevine plants aim at protecting the integrity of water transport pathway by closing stomata before embolism occurs in stems, this finding suggests that genotypes that are inherently more vulnerable to embolism than others may also display earlier stomatal closure, thus reduced gas exchange rates and productivity, under more stressful conditions. Moreover, wine producing regions bearing few varieties with high embolism vulnerability might be particularly at risk to undergo large-scale events of hydraulic failure in vineyards under sustained drought periods, regardless of environmental conditions of regions and water management strategies. This study outlines the importance of accounting for hydraulic traits related to plant drought resistance to recommend grapevine varieties adapted to local climate and improve viticulture suitability in the context of climate change.

## Material and methods

### Plant material

All plants used were *Vitis* rooted cuttings that were placed during the 2015–2018 study period under the same glasshouse, located at the Nouvelle-Aquitaine Bordeaux research center of the National Research Institute for Agriculture, Food and Environment (INRAE), France (44°47′23.8′′ N, 0°34′39.3″ W). Cuttings were prepared from dormant canes that were collected in vineyards of the research center (Supplementary Table S1). They were grown in 7-L pots filled with 1 kg of gravel and 5.5 kg of commercial potting medium (70% horticultural substrate and 30% sand), and kept well-watered until measurements. They were drip irrigated with a nutrient solution (0.1 mM NH_4_H_2_PO_4_, 0.187 mM NH_4_NO_3_, 0.255 mM KNO_3_, 0.025 mM MgSO_4_, 0.002 mM Fe, and oligo-elements [B, Zn, Mn, Cu, and Mo]) in order to prevent any mineral deficiency during plant development. The surface of each pot was covered with burlap to avoid water loss by soil evaporation.

Measurements were conducted on 240 plants from 30 *Vitis* genotypes (Supplementary Table S1), encompassing (*i*) 22 *Vitis vinifera* L. varieties; (*ii*) five interspecific hybrid varieties (Artaban, Floreal, Regent, Vidoc and Voltis) characterized by a polygenic resistance to powdery and downy mildews^75^, and (*iii*) three commonly used *Vitis* rootstocks (Richter 110 (110R; *V*. *berlandieri* x *V*. *rupestris*), Riparia ‘Gloire de Montpellier’ (RGM; *V*. *riparia*) and Selektion Oppenheim 4 (SO4; *V*. *berlandieri* x *V*. *riparia*)). The 22 *Vitis vinifera* varieties, including 11 black-berried and 11 white-berried varieties, spanned three *Vitis vinifera* subspecies (12 *Vitis vinifera* subsp. (proles) occidentalis, seven subsp. (proles) orientalis, and three subsp. (proles) pontica) and four geographical groups (two varieties from Balkans, three from Eastern Mediteranean and Caucasus, two from the Iberian Peninsula, and 15 from Western and Central Europe)^76,77^. Accounting for ~ 1,9 M hectares cultivated, they represented > 42% of the global winegrape bearing area (data from https://www.adelaide.edu.au/press/titles/winegrapes). The study included in particular nine of the top 10 winegrape varieties, namely Cabernet Sauvignon, Merlot, Tempranillo, Chardonnay, Syrah, Grenache, Sauvignon blanc, Ugni blanc and Pinot noir.

### Vulnerability to xylem embolism

In situ* flow-centrifuge technique (MEGA-CAVITRON).*

Stem vulnerability to xylem embolism was assessed using 220 plants. The variability among species and varieties was characterized by measuring 176 one-year-old plants in September and early October after shoot hardening (c.f ‘summer’ throughout the manuscript; Supplementary Table S2). The variability within variety was elucidated in two ways: (i) within a single growing season for six varieties, using additional 39 one-year-old plants that were measured in July before shoot hardening (c.f ‘spring’); and (ii) across seasons in Syrah, by carrying out further measurements in spring of the following year on current-year shoots of five two-year-old plants coming from the same initial cohort. The screening campaigns spread out across growing seasons (from 2015 to 2018), with 90% of varieties measured during a single season. There was no variability in the resistance of xylem embolism between years for the varieties measured during multiple seasons (*P* > 0.05 in all cases), and we are thus confident that the comparisons among varieties reported in this study are accurate.

Measurements were carried out using the Cavitron technique^34^ at the high-throughput phenotyping platform for hydraulic traits (Caviplace, Phenobois platform, University of Bordeaux, Pessac, France). They were conducted using a 100-cm diameter rotor (DG-MECA, Gradignan, France) that has allowed artefact-free embolism resistance determination in long-vesseled species for which the use of standard (27-cm large rotor) and large (42-cm large) Cavitrons proved to be unsuccessful in determining maximum stem hydraulic conductivity^18,37^. Plants were brought from the glasshouse to the laboratory early in the morning of each measurement day, entirely conditioned with plastic bags to avoid water loss by transpiration and high xylem tensions that could induce artifactual xylem embolism. The main stems were then kept in water for relaxation during ca. 1 h prior to measurements, when they were cut under water to a standard length of 1 m. A solution of ultrapure and deionized water containing 10 mM KCl and 1 mM CaCl_2_ was used as reference ionic solution, while the centrifuge rotation speed was initially set to induce a xylem pressure of − 0.8 MPa before being gradually increased to lower xylem pressures by − 0.5 MPa. Hydraulic conductivities at every rotation speed (*k*_i_, kg m^−1^ s^−1^ MPa^−1^) were measured using the Cavisoft software (v. 5.2, University of Bordeaux). The percentage loss of hydraulic conductivity (PLC) was determined at each pressure as follows:

1 $$PLC \, = \, 100*\left( {1 - k_{i} /k_{\max } } \right)$$

where *k*_max_ represents the maximum hydraulic conductivity measured at the first induced xylem pressure (i.e. − 0.8 MPa).

#### Optical vulnerability (OV) technique

Intra-variability in leaf vulnerability to embolism was characterized by studying xylem embolism patterns in basal and apical leaves during both spring and summer 2019. Eight and six one-year-old Cabernet Sauvignon plants were used in spring and summer, respectively. Additional measurements were carried out in spring using six one-year-old Syrah plants. Plants, which were left unpruned, did not differ in stem diameter nor in height between seasons (8.8 ± 0.2 mm vs. 8.5 ± 0.6 mm large, and 256.6 ± 8.6 cm vs. 265.1 ± 20.8 cm tall in spring and summer, respectively). They also had the same number of leaves (96 ± 5 vs 87 ± 10 leaves in spring and summer, respectively). Basal and apical leaves were taken from the 8th–14th and 80th–90th nodes, respectively.

All plants were kept well-hydrated prior to measurements and were brought from the glasshouse to the laboratory early in the morning of each measurement day to avoid water loss by transpiration. The plastic bags covering plants were removed once the scanned leaves and stem psychrometers (see below) were installed. Visualization of the onset and accumulation of embolism in leaves was carried out using the optical technique^35^ (see also http://www.opensourceov.org for detailed instructions). In each individual, one mature basal leaf and one mature apical leaf, which were still attached to the parent grapevine, were placed on two different scanners (Perfection V800 Photo, EPSON, Suwa, Japan) located in a dark room with controlled conditions at 26 °C and 50% humidity. They were fixed with a transparent glass and adhesive tape to avoid as much leaf shrinkage as possible during plant desiccation. Scan magnification was set with the Epson scanner software to give sufficient resolution of midrib and other order veins. The following settings were selected: film (with film area guide) as document type, color positive film as document source, 8-bit grayscale as image type, and a 2400 dpi resolution. Brightness and contrasts as well as leaf scanned area were adjusted to optimize visualization of embolism events and provide images not exceeding 9 Mb each. Each leaf was automatically scanned every five minutes using the AutoIt automation software until mesophyll cells turned from green to brown, which indicated cell death. Complete plant dehydration in spring and summer was observed after ca. 80 h and 130 h of scanning, respectively.

Stem water potentials (*Ψ*_stem_) were continuously recorded throughout plant dehydration using stem psychrometers (ICT International, Armidale, NSW, Australia). We first examined the homogeneity in water potential along the plant stem by installing in two plants two psychrometers that were located below the base and top scanned leaves, respectively. As there was a good agreement in water potential between the stem base and top (Supplementary Fig. S7a), one psychrometer per plant was then used for the other grapevines and installed halfway up the main stem. *Ψ*_stem_ values were automatically recorded every 30 min. The accuracy of psychrometer readings was regularly cross-validated by pressure bomb measurements (DG-MECA, Gradignan, France), using adjacent leaves that had been covered for at least two hours with aluminium foil and wrapped in a plastic bag (Supplementary Fig. S7b).

Upon completion, the stacks of captured images, which comprised between 1400 and 2200 scans, were analyzed using ImageJ software^78^ in order to reveal embolism events (i.e. air entry into leaf xylem vessels), that corresponded to rapid changes in light transmission through leaf veins (see https://www.opensourceov.org/ for a detailed description of the analysis process). Briefly, an image subtraction method was used to emphasize changes in light transmission between successive images of a given stack of images. The new stack of subtracted images was then thresholded to reveal embolism events, appearing as white pixels on a dark background, before the ‘Analyze Particles’ function was used to filter out noise, corresponding to small changes small random changes in pixel contrast compared to large, structured embolism events^35^, and automatically count the number of embolized pixels observed in leaf veins. All embolized pixels recorded throughout plant dehydration were then summed up to determine a time-based accumulation of embolism. These data were then combined with the water potential timeline to determine stem water potential associated with each embolism event. An optical vulnerability curve was determined by representing the accumulation of embolized pixels as a function of *Ψ*_leaf_ (see the following section).

#### Vulnerability curve fitting

Vulnerability curves, corresponding to percentage loss of hydraulic conductivity (PLC, %) as a function of xylem pressure (for the centrifugation technique) or percentage of embolized pixels (PEP, %) as a function of stem water potential (for the optical vulnerability technique), were fitted using the NLIN procedure in SAS 9.4 (SAS, Cary, NC, USA) based on the following equation^79^:

2 $${\text{PLC or PEP}} = 100/\left( {1 + \exp \left( {S/25*\left( {\Psi - \Psi_{50} } \right)} \right)} \right)$$

where *Ψ*_50_ (MPa) is the xylem pressure/water potential inducing 50% loss of hydraulic conductivity and *S* (% MPa^-1^) is the slope of the vulnerability curve at the inflexion point. The xylem pressures/water potentials inducing 12% (*Ψ*_12_) and 88% (*Ψ*_88_) loss of hydraulic conductivity were calculated as follows: *Ψ*_12_ = 50/*S* + *Ψ*_50_ and *Ψ*_88_ = -50/*S* + *Ψ*_50_. One vulnerability curve was obtained per plant in both centrifuge and optical measurements. In other words, the Pammenter model was fitted on each vulnerability curve to obtain a *Ψ*_50_ and slope values per leaf and/or individual, and subsequently an average value per *Vitis* leaf location, variety and season.

### Hydraulic vulnerability segmentation (HVS)

Vulnerability to xylem embolism was compared between leaves and stems in Cabernet Sauvignon and Syrah by calculating the differences between organs in water potentials inducing 12% and 50% embolism. This was done in both spring and summer and for both basal and apical leaves of each plant measured with the OV technique, using the mean stem *Ψ*_12_ and *Ψ*_50_ per variety and season from the flow-centrifuge technique measurements. The resulting terms, respectively named HVS_Ψ12_ and HVS_Ψ50_, aimed at reflecting the extent to which leaves act as hydraulic fuses to protect perennial organs (i.e. stems) during prolonged drought.

### Xylem anatomy

Xylem anatomical observations were carried out in 19 varieties (15 *Vitis vinifera* and the four hybrid varieties; Supplementary Table S10) from stem pieces that were collected during the centrifugation measurement campaign of summer 2018. Stem pieces were sampled in the middle of the 1-m centrifuged branches (*n* = 50), and placed in 80% ethyl-alcohol. Stem cross-sections  (50 µm thick, bark removed) were made using a GSL1 microtome^80^, stained using a 1% safranin O solution (96% ethyl-alcohol), rinsed twice in 100% ethyl-alcohol and transferred in xylene. Slices were then mounted between slide and coverslip in resin (Histolaque LMR, Labo-Moderne, Gennevilliers, France). High resolution micrographs (~ 500 nm/pixel) were obtained at the Bordeaux Imaging Center (a member of the France Bio-Imaging national infrastructure, ANR-10-INBS-04) using a NANOZOOMER 2.0HT (Hamamatsu Photonics, Hamamatsu City, Japan) in brightfield mode. Xylem vessel areas were measured in the total area of each cross section using ImageJ software^78^. The following anatomical traits were calculated following Scholz et al.^81^: equivalent circle diameter (*D*, in µm), weighted hydraulic diameter (*D*_H_, in µm), xylem area (*A*_xyl_, in m^2^) and xylem vessel density (*V*_D_, number of vessels per mm^2^). The theoretical specific hydraulic conductivity *(k*_th_, in kg s^−1^ m^−1^ MPa^−1^) was calculated using the Hagen-Poiseuille equation:

3 $$k_{th} = \sum ((\pi *D^{4} *\rho )/\left( {128*\eta } \right))*1/A_{xyl}$$

where *D* is the equivalent circle diameter (in m), *ρ* the density of water (998.2 kg m^−3^ at 20 °C), *η* the viscosity of water (1.002 10^–9^ MPa s at 20 °C) and *A*_xyl_ the xylem area (in m^2^).

### Regional risk index of drought vulnerability

A risk index of vulnerability to drought was calculated per wine producing region or country by coupling winegrape varietal coverage data with summer stem *Ψ*_50_ values of *Vitis vinifera* varieties. First, information on *Vitis vinifera* varietal coverage were retrieved from the online database of regional and national winegrape bearing areas by variety^72^ (https://economics.adelaide.edu.au/wine-economics/databases#database-of-regional-national-and-global-winegrape-bearing-areas-by-variety-1960-to-2016). The dataset we compiled provided varietal coverage of 22 *Vitis* varieties (all the *Vitis vinifera* studied but Müsküle, for which online data were not available, as well as Regent), for a total of 653 wine regions across 48 countries (Supplementary Data S1, Supplementary Fig. S6a,b). Second, this dataset was used to calculate, for each wine region or country, the bearing area of the four clusters of vulnerability to xylem embolism (low, low-to-medium, medium-to-high, high) that were previously identified from the hierarchical clustering analysis (see the Statistical analyses section; Supplementary Fig. S6c,d). Third, each cluster was assigned a specific weight (or score) on a 0–1 scale of vulnerability to xylem embolism: (i) low vulnerability: range 0–0.25 (median = 0.125); (ii) low-to-medium vulnerability: range 0.25–0.50 (median = 0.375); (iii) medium-to-high vulnerability: range 0.50–0.75 (median = 0.625); (iv) high vulnerability: range 0.75–1 (median = 0.875). The risk index (RI), accounting for both the bearing area and the vulnerability to xylem embolism of each cluster, was then calculated as follows (Supplementary Data S2):

4 $$\begin{aligned} {\text{RI }} & = \, 0.125.{\text{bearing area}}\left[ {{\text{low}}} \right] \, + \, 0.375.{\text{bearing area}}\left[ {\text{low - to - medium}} \right] \\ & \quad + \, 0.625.{\text{bearing area}}\left[ {\text{medium - to - high}} \right] \, + \, 0.875.{\text{bearing area}}\left[ {{\text{high}}} \right] \\ \end{aligned}$$

where each bearing area is represented as a percent of the regional area.

This risk index was mapped for 329 regions including 13 countries for which our varietal coverage dataset represented at least 40% of the regional or national winegrape bearing area. Thus, we provided a risk index for 52% of the global wine producing regions.

### Statistical analyses

Overall differences in summer stem vulnerability to xylem embolism (*Ψ*_12_, *Ψ*_50_, *Ψ*_88_ and *S*) and xylem anatomical traits (*A*_xyl_, *D*, *D*_H_, *k*_th_ and *V*_D_) among the 30 grapevine genotypes were examined with one-way analyses of variance and Student–Newman–Keuls post hoc tests (ANOVA procedure in SAS, version 9.4, SAS Institute, Cary, NC, USA). A hierarchical clustering analysis was performed based on the varietal *Ψ*_12_, *Ψ*_50_, *Ψ*_88_ values, using the Euclidean distance matrix and Ward criterion for clustering from the ‘heatmap’ package in RStudio v1.4.

Differences in summer stem vulnerability to xylem embolism and xylem anatomical traits among types of varieties (*Vitis vinifera* vs hybrids vs rootstocks) as well as differences in summer stem vulnerability to xylem embolism among *Vitis vinifera* subspecies (proles occidentalis vs proles orientalis vs proles pontica), geographical origins (Balkans vs Eastern Mediterranean & Caucasus vs Iberian Peninsula vs Western & Central Europe) and between berry skin colors (black vs white) were studied with generalized linear models (GLM procedure in SAS), where type of varieties/geographical origin/berry skin color was treated as a fixed factor and variety nested within type of varieties/geographical origin/berry skin color as a random factor.

Intra-variety seasonality in stem vulnerability to embolism, i.e. the difference in embolism vulnerability between spring and summer (in 110R, Cabernet Sauvignon, Grenache, Merlot, Regent and Syrah) as well as the difference in spring embolism vulnerability between one- and two-year-old plants (in Syrah), was assessed with Student’s t-tests (TTEST procedure in SAS). Intra-variety seasonality in leaf vulnerability to embolism and the difference in hydraulic vulnerability segmentation were tested in Cabernet Sauvignon using a generalized linear model where season (spring vs summer), leaf location (basal vs apical) and the interaction season*leaf location were treated as fixed effects, and in Syrah. Differences in vulnerability to embolism and hydraulic vulnerability segmentation between basal and apical leaves of Syrah plants measured in spring were tested using Student’s t-tests.

Relationships between hydraulic and anatomical traits were explored using a generalized linear mixed model (GLIMMIX procedure in SAS) where variety was treated as a random factor. Normality of data and homogeneity of variances were assessed prior to all analyses.

### Plant ethics

Experimental research and field studies on plants including the collection of plant material, comply with relevant institutional, national, and international guidelines and legislation.

## Supplementary Information

Supplementary Information 1.

Supplementary Information 2.

Supplementary Information 3.

## Data Availability

The datasets used during the current study are available from the corresponding author on reasonable request.
