# Supplementary material for: Quantifying the grapevine xylem embolism resistance spectrum to identify varieties and regions at risk in a future dry climate
Source: Sci Rep. 2023 May 12;13:7724. doi: 10.1038/s41598-023-34224-6 (PMC10181993; doi:10.1038/s41598-023-34224-6)
Supplement: Supplementary file 1 — Supplementary Information 1. [file 41598_2023_34224_MOESM1_ESM.docx]

Supplementary Information for

**Quantifying the grapevine xylem embolism resistance spectrum to identify varieties and regions at risk in a future dry climate**

Laurent J. Lamarque *et al.*

*Corresponding author. Email: [llamarqueab@gmail.com](mailto:llamarqueab@gmail.com)

**This PDF file includes:**

Supplementary Fig. S1 to S7

Supplementary Table S1 to S12

**Other Supplementary Information for this manuscript include the following:**

Supplementary Data S1 to S2

**Supplementary Table S1.** **Characteristics of grapevines included in the study.** *V*IVC refers to the *Vitis* International Variety Catalogue (<https://www.vivc.de/>). *Vitis* rootstocks 110R, RGM, and SO4 correspond to Richter 110, *Vitis riparia* ‘Gloire de Montpellier’ and Selektion Oppenheim 4, respectively. Region refers to the geographic origins as follows: BALK: Balkans, EMCA: Eastern Mediterranean & Caucasus, IBER: Iberian Peninsula, WCEUR: Western & Central Europe. Skin refers to the berry skin color. Information about country of origin was retrieved from the VIVC, and that about *Vitis vinifera* subspecies (proles), region, and berry skin color from Bacilieri *et al.* (2013) and Negrul (1946) [76, 77]. Plant material has been collected in an experimental vineyard (VitAdapt) or grapevine collections at the Nouvelle-Aquitaine Bordeaux research center of the National Research Institute for Agriculture, Food and Environment (INRAE), France (44°47’23.8’’N, 0°34’39.3”W).

|  |  |  | Geographic origin | |  | Material source |
| --- | --- | --- | --- | --- | --- | --- |
| Variety | *V*IVC # | Subspecies | Country | Region | Skin |  |
| *Vitis vinifera* |  |  |  |  |  |  |
| Cabernet franc | 1927 | occidentalis | France | WCEUR | Black | VitAdapt |
| Cabernet Sauvignon | 1929 | occidentalis | France | WCEUR | Black | VitAdapt |
| Carignan | 2098 | occidentalis | France | WCEUR | Black | Collections |
| Chardonnay | 2455 | occidentalis | France | WCEUR | White | VitAdapt |
| Cinsaut | 2672 | occidentalis | France | WCEUR | Black | Collections |
| Clairette | 2695 | occidentalis | France | WCEUR | White | Collections |
| Dabouki | 3309 | orientalis | Israel | EMCA | White | Collections |
| Grenache | 4461 | orientalis | Spain | WCEUR | Black | VitAdapt |
| Merlot | 7657 | occidentalis | France | WCEUR | Black | VitAdapt |
| Muscat Ottonel | 8243 | pontica | France | WCEUR | White | Collections |
| Müsküle | 8146 | orientalis | Turkey | EMCA | White | Collections |
| Perle de Csaba | 9166 | pontica | Hungary | BALK | White | Collections |
| Pinot noir | 9279 | occidentalis | France | WCEUR | Black | VitAdapt |
| Riesling | 10077 | occidentalis | Germany | WCEUR | White | Collections |
| Saperavi | 10708 | orientalis | Georgia | EMCA | Black | Collections |
| Sauvignon blanc | 10790 | occidentalis | France | WCEUR | White | VitAdapt |
| Sultanine (Thompson Seedless) | 12051 | orientalis | Turkey | BALK | White | Collections |
| Sylvaner | 11805 | occidentalis | Austria | WCEUR | White | Collections |
| Syrah | 3738 | occidentalis | France | WCEUR | Black | VitAdapt |
| Tempranillo | 12350 | orientalis | Spain | IBER | Black | VitAdapt |
| Tinta pinheira | 10331 | orientalis | Portugal | IBER | Black | VitAdapt |
| Ugni blanc | 12628 | pontica | Italy | WCEUR | White | Collections |
| *Hybrid varieties* |  |  |  |  |  |  |
| Artaban (Bouquet x Regent) | 25804 | - | France | - | Black | Collections |
| Floreal (Bouquet x Villaris) | 25805 | - | France | - | White | Collections |
| Regent (Diana x Chambourcin) | 4572 | - | Germany | - | Black | Collections |
| Vidoc (Bouquet x Regent) | 25806 | - | France | - | Black | Collections |
| Voltis (Bouquet x Villaris) | 25807 | - | France | - | White | Collections |
| *Rootstocks* |  |  |  |  |  |  |
| 110R (*V*. *berlandieri* x *V*. *rupestris*) | 10065 | - | France | - | - | Collections |
| RGM (*V*. *riparia*) | 4824 | - | France | - | - | Collections |
| SO4 (*V*. *berlandieri* x *V*. *riparia*) | 11473 | - | Germany | - | - | Collections |

**Supplementary Table S2. Stem vulnerability to xylem embolism over the course of the growing season.** Spring and summer mean values (± standard errors) of xylem pressure inducing 12 %, 50 %, and 88 % loss of hydraulic conductivity (*Ψ*_12_, *Ψ*_50_, and *Ψ*_88_, respectively; in MPa), and of the slope of the vulnerability curve at the inflection point (*S*; in % MPa^-1^) in four *Vitis vinifera* varieties (Cabernet Sauvignon, Grenache, Merlot, and Syrah), one hybrid variety (Regent), and one rootstock (110R). Measurements were carried out on one-year-old plants. Numbers in brackets refer to sample sizes. *F* values refer to t-tests conducted between seasons per variety. **P* < 0.05.

|  | Spring | Summer |  | Season | |
| --- | --- | --- | --- | --- | --- |
|  |  |  |  | *df* | *F* |
| *110R* |  |  |  |  |  |
| *Ψ*_12_ | -1.45 ± 0.19 (3) | -1.83 ± 0.22 (5) |  | 5 | 0.99 |
| *Ψ*_50_ | -2.29 ± 0.21 (3) | -3.15 ± 0.11 (5) |  | 5 | 3.88* |
| *Ψ*_88_ | -3.13 ± 0.23 (3) | -4.48 ± 0.13 (5) |  | 5 | 5.30* |
| *S* | 59.54 ± 1.41 (3) | 39.32 ± 3.79 (5) |  | 5 | 3.17* |
| *Cabernet Sauvignon* | |  |  |  |  |
| *Ψ*_12_ | 1.75 ± 0.08 (5) | -2.19 ± 0.15 (14) |  | 17 | 2.56* |
| *Ψ*_50_ | -2.49 ± 0.08 (5) | -3.07 ± 0.10 (14) |  | 17 | 3.37* |
| *Ψ*_88_ | -3.23 ± 0.12 (5) | -3.94 ± 0.13 (14) |  | 17 | 3.15* |
| *S* | 69.81 ± 7.14 (5) | 66.03 ± 6.16 (14) |  | 17 | 0.34 |
| *Grenache* |  |  |  |  |  |
| *Ψ*_12_ | -1.38 ± 0.10 (9) | -1.85 ± 0.08 (23) |  | 30 | 3.23* |
| *Ψ*_50_ | -2.14 ± 0.09 (9) | -2.69 ± 0.04 (23) |  | 30 | 6.21* |
| *Ψ*_88_ | -2.91 ± 0.11 (9) | -3.53 ± 0.07 (23) |  | 30 | 4.73* |
| *S* | 67.92 ± 4.76 (9) | 69.07 ± 6.24 (23) |  | 30 | -0.15 |
| *Merlot* |  |  |  |  |  |
| *Ψ*_12_ | -1.79 ± 0.08 (6) | -1.81 ± 0.26 (9) |  | 13 | 0.06 |
| *Ψ*_50_ | -2.52 ± 0.09 (6) | -2.97 ± 0.12 (9) |  | 13 | 2.67* |
| *Ψ*_88_ | -3.25 ± 0.14 (6) | -4.14 ± 0.10 (9) |  | 13 | 5.48* |
| *S* | 72.03 ± 7.08 (6) | 53.78 ± 11.56 (9) |  | 13 | 1.18 |
| *Regent* |  |  |  |  |  |
| *Ψ*_12_ | -1.61 ± 0.16 (5) | -1.57 ± 0.14 (6) |  | 9 | -0.18 |
| *Ψ*_50_ | -2.58 ± 0.08 (5) | -2.76 ± 0.13 (6) |  | 9 | 1.08 |
| *Ψ*_88_ | -3.55 ± 0.11 (5) | -3.95 ± 0.21 (6) |  | 9 | 1.56 |
| *S* | 54.35 ± 6.69 (5) | 43.93 ± 3.91 (6) |  | 9 | 1.40 |
| *Syrah* |  |  |  |  |  |
| *Ψ*_12_ | -1.51 ± 0.09 (11) | -2.13 ± 0.07 (22) |  | 31 | 5.51* |
| *Ψ*_50_ | -2.26 ± 0.06 (11) | -2.90 ± 0.05 (22) |  | 31 | 7.80* |
| *Ψ*_88_ | -3.01 ± 0.05 (11) | -3.66 ± 0.09 (22) |  | 31 | 6.27* |
| *S* | 68.57 ± 2.91 (11) | 76.49 ± 7.36 (22) |  | 31 | -1.00 |

**Supplementary Table S3. Stem vulnerability to xylem embolism over the years.** Spring mean values (± standard errors) of xylem pressure inducing 12 %, 50 %, and 88 % loss of hydraulic conductivity (*Ψ*_12_, *Ψ*_50_, and *Ψ*_88_, respectively; in MPa), and of the slope of the vulnerability curve at the inflection point (*S*; in % MPa^-1^) in one- and two-year-old Syrah plants. Numbers in brackets refer to sample sizes. *F* values refer to t-tests conducted between ages. **P* < 0.05.

| Trait | Spring year 1 | Spring year 2 |  |  | Age |
| --- | --- | --- | --- | --- | --- |
|  |  |  |  | *df* | *F* |
| *Syrah* |  |  |  |  |  |
| *Ψ*_12_ | -1.54 ± 0.11 (8) | -1.70 ± 0.15 (5) |  | 11 | 0.85 |
| *Ψ*_50_ | -2.32 ± 0.07 (8) | -2.68 ± 0.09 (5) |  | 11 | 3.10* |
| *Ψ*_88_ | -3.11 ± 0.06 (8) | -3.67 ± 0.15 (5) |  | 11 | 3.97* |
| *S* | 65.06 ± 3.40 (8) | 54.53 ± 7.30 (5) |  | 11 | 1.48 |

**Supplementary Table S4. Leaf vulnerability to xylem embolism over the course of the growing season.** Spring and summer mean values (± standard errors) of water potentials inducing 12 %, 50 %, and 88 % loss of hydraulic conductivity (*Ψ*_12_, *Ψ*_50_, and *Ψ*_88_, respectively; in MPa), and of the slope of the vulnerability curve at the inflection point (*S*; in % MPa^-1^) and the hydraulic vulnerability segmentation (HVS*_Ψ_*_12_ and HVS*_Ψ_*_50_; in MPa) for basal and apical leaves of Cabernet Sauvignon and Syrah plants. Measurements were carried out on one-year-old plants. HVS*_Ψ_*_12_ and HVS*_Ψ_*_50_ were calculated as *Ψ*_12_(leaf)-*Ψ*_12_(stem) and *Ψ*_50_(leaf)-*Ψ*_50_(stem), respectively, with stem *Ψ*_12_ and *Ψ*_50_ values taken from the flow-centrifugation measurements (i.e. one mean value per species per season). Numbers in brackets refer to sample sizes. *F* values refer to generalized linear models (in Cabernet Sauvignon) and t-tests (in Syrah). **P* < 0.05.

| Trait |  | Spring | |  | Summer | |  | Season | |  | Leaf | |  | Season x leaf | |
| --- | --- | --- | --- | --- | --- | --- | --- | --- | --- | --- | --- | --- | --- | --- | --- |
|  |  | Basal leaf | Apical leaf |  | Basal leaf | Apical leaf |  | *df* | *F* |  | *df* | *F* |  | *df* | *F* |
| *Cabernet Sauvignon* | | |  |  |  |  |  |  |  |  |  |  |  |  |  |
| *Ψ*_12_ |  | -1.51 ± 0.09 (7) | -1.42 ± 0.17 (8) |  | -1.65 ± 0.12 (6) | -1.79 ± 0.14 (6) |  | 1 | 3.54 |  | 1 | 0.03 |  | 1 | 0.74 |
| *Ψ*_50_ |  | -1.74 ± 0.10 (7) | -1.91 ± 0.13 (8) |  | -1.92 ± 0.11 (6) | -2.17 ± 0.18 (6) |  | 1 | 2.71 |  | 1 | 2.47 |  | 1 | 0.08 |
| *Ψ*_88_ |  | -1.98 ± 0.14 (7) | -2.41 ± 0.16 (8) |  | -2.19 ± 0.13 (6) | -2.54 ± 0.25 (6) |  | 1 | 0.99 |  | 1 | 4.91* |  | 1 | 0.06 |
| *S* |  | 273.32 ± 40.36 (7) | 144.99 ± 33.72 (8) |  | 224.62 ± 41.35 (6) | 157.98 ± 24.67 (6) |  | 1 | 0.24 |  | 1 | 7.21* |  | 1 | 0.72 |
| HVS*_Ψ_*_12_ |  | 0.24 ± 0.09 (7) | 0.33 ± 0.17 (8) |  | 0.54 ± 0.12 (6) | 0.40 ± 0.14 (6) |  | 1 | 1.78 |  | 1 | 0.03 |  | 1 | 0.74 |
| HVS*_Ψ_*_50_ |  | 0.75 ± 0.10 (7) | 0.58 ± 0.13 (8) |  | 1.14 ± 0.11 (6) | 0.90 ± 0.18 (6) |  | 1 | 7.42* |  | 1 | 2.47 |  | 1 | 0.08 |
| *Syrah* | |  |  |  |  |  |  |  |  |  |  |  |  |  |  |
| *Ψ*_12_ |  | -1.33 ± 0.08 (6) | -1.32 ± 0.12 (6) |  | - | - |  |  | - |  | 10 | -0.04 |  |  | - |
| *Ψ*_50_ |  | -1.49 ± 0.10 (6) | -1.80 ± 0.15 (6) |  | - | - |  |  | - |  | 10 | 1.69 |  |  | - |
| *Ψ*_88_ |  | -1.66 ± 0.12 (6) | -2.27 ± 0.21 (6) |  | - | - |  |  | - |  | 10 | 2.51* |  |  | - |
| *S* |  | 355.55 ± 64.82 (6) | 129.56 ± 26.64 (6) |  | - | - |  |  | - |  | 10 | 3.22* |  |  | - |
| HVS*_Ψ_*_12_ |  | 0.18 ± 0.08 (6) | 0.19 ± 0.12 (6) |  | - | - |  |  | - |  | 10 | -0.04 |  |  | - |
| HVS*_Ψ_*_50_ |  | 0.77 ± 0.10 (6) | 0.47 ± 0.15 (6) |  | - | - |  |  | - |  | 10 | 1.69 |  |  | - |

**Supplementary Table S5.** **Xylem embolism resistance spectrum in grapevine.** Mean values (± standard errors) of xylem pressure inducing 12 %, 50 %, and 88 % loss of hydraulic conductivity (*Ψ*_12_, *Ψ*_50,_ and *Ψ*_88_, respectively; in MPa), and of the slope of the vulnerability curve at the inflection point (*S*; in % MPa^-1^) for 22 *Vitis vinifera* varieties, 5 hybrid varieties, and 3 rootstocks. Measurements were carried out in summer on one-year-old plants. Numbers in brackets refer to sample sizes.

| Variety | *Ψ*_12_ | *Ψ*_50_ | *Ψ*_88_ | *S* |
| --- | --- | --- | --- | --- |
| *Vitis vinifera* |  |  |  |  |
| Cabernet franc (3) | -0.81 ± 0.13 | -2.51 ± 0.08 | -4.22 ± 0.03 | 29.28 ± 0.83 |
| Cabernet Sauvignon (14) | -2.19 ± 0.15 | -3.07 ± 0.10 | -3.94 ± 0.13 | 66.03 ± 6.16 |
| Carignan (5) | -2.07 ± 0.07 | -2.57 ± 0.07 | -3.08 ± 0.06 | 100.16 ± 4.07 |
| Chardonnay (3) | -1.30 ± 0.26 | -2.50 ± 0.09 | -3.71 ± 0.08 | 43.42 ± 7.23 |
| Cinsaut (7) | -1.41 ± 0.11 | -2.58 ± 0.07 | -3.75 ± 0.07 | 43.47 ± 2.31 |
| Clairette (3) | -1.38 ± 0.02 | -2.48 ± 0.04 | -3.57 ± 0.06 | 45.65 ± 0.85 |
| Dabouki (5) | -1.63 ± 0.13 | -2.43 ± 0.14 | -3.23 ± 0.19 | 64.54 ± 5.63 |
| Grenache (23) | -1.85 ± 0.08 | -2.69 ± 0.04 | -3.53 ± 0.07 | 69.07 ± 6.24 |
| Merlot (9) | -1.81 ± 0.26 | -2.97 ± 0.12 | -4.14 ± 0.10 | 53.78 ± 11.56 |
| Muscat Ottonel (5) | -2.32 ± 0.08 | -2.82 ± 0.04 | -3.32 ± 0.05 | 106.07 ± 14.18 |
| Müsküle (4) | -1.93 ± 0.20 | -2.80 ± 0.11 | -3.67 ± 0.07 | 59.81 ± 6.98 |
| Perle de Csaba (5) | -2.14 ± 0.10 | -2.79 ± 0.08 | -3.43 ± 0.06 | 77.57 ± 3.11 |
| Pinot noir (3) | -2.71 ± 0.12 | -3.27 ± 0.02 | -3.83 ± 0.07 | 91.37 ± 14.87 |
| Riesling (5) | -1.95 ± 0.24 | -2.74 ± 0.08 | -3.53 ± 0.22 | 84.05 ± 19.44 |
| Saperavi (8) | -1.60 ± 0.13 | -2.52 ± 0.06 | -3.43 ± 0.05 | 57.99 ± 5.65 |
| Sauvignon blanc (3) | -1.71 ± 0.91 | -2.74 ± 0.38 | -3.77 ± 0.14 | 65.83 ± 33.68 |
| Sultanine (4) | -0.91 ± 0.16 | -1.91 ± 0.09 | -2.92 ± 0.22 | 54.49 ± 10.01 |
| Sylvaner (4) | -2.00 ± 0.26 | -2.97 ± 0.16 | -3.94 ± 0.07 | 53.10 ± 4.84 |
| Syrah (22) | -2.13 ± 0.07 | -2.90 ± 0.05 | -3.66 ± 0.09 | 76.49 ± 7.36 |
| Tempranillo (3) | -1.92 ± 0.21 | -2.76 ± 0.12 | -3.60 ± 0.07 | 61.73 ± 8.76 |
| Tinta pinheira (3) | -1.37 ± 0.26 | -2.72 ± 0.04 | -4.08 ± 0.35 | 38.80 ± 8.63 |
| Ugni blanc (3) | -1.49 ± 0.32 | -2.44 ± 0.18 | -3.39 ± 0.05 | 55.10 ± 9.05 |
| *Hybrid varieties* |  |  |  |  |
| Artaban (3) | -1.64 ± 0.22 | -2.82 ± 0.15 | -3.99 ± 0.09 | 42.71 ± 2.37 |
| Floreal (3) | -0.37 ± 0.12 | -1.79 ± 0.21 | -3.21 ± 0.31 | 35.43 ± 2.35 |
| Regent (6) | -1.57 ± 0.14 | -2.76 ± 0.13 | -3.95 ± 0.21 | 43.93 ± 3.91 |
| Vidoc (3) | -0.47 ± 0.51 | -2.00 ± 0.17 | -3.53 ± 0.17 | 34.30 ± 7.58 |
| Voltis (3) | -1.21 ± 0.32 | -2.13 ± 0.21 | -3.04 ± 0.10 | 56.75 ± 7.73 |
| *Rootstocks* |  |  |  |  |
| 110R (5) | -1.83 ± 0.22 | -3.15 ± 0.11 | -4.48 ± 0.13 | 39.32 ± 3.79 |
| RGM (5) | -1.74 ± 0.19 | -3.05 ± 0.14 | -4.36 ± 0.14 | 38.90 ± 2.75 |
| SO4 (6) | -1.76 ± 0.20 | -3.36 ± 0.11 | -4.96 ± 0.12 | 32.24 ± 2.47 |

**Supplementary Table S6. Difference in stem vulnerability to xylem embolism among types of grapevine.** Summer mean values (± standard errors) of xylem pressure inducing 12 %, 50 %, and 88 % loss of hydraulic conductivity (*Ψ*_12_, *Ψ*_50,_ and *Ψ*_88_, respectively; in MPa), and of the slope of the vulnerability curve at the inflection point (*S*; in % MPa^-1^) in *Vitis vinifera* varieties, hybrid varieties, and rootstocks. Measurements were carried out on one-year-old plants. *n* = 139, 15, and 16 plants for *Vitis vinifera* varieties, hybrid varieties, and rootstocks, respectively. *F* values refer to generalized linear models where type of varieties was treated as a fixed factor and variety nested within type of variety as a random factor. **P* < 0.05. Letters refer to Student-Newman-Keuls post hoc tests. Different letters mean statistically significant differences.

| Trait | *Vitis vinifera* | Hybrids | Rootstocks |  |  | Type |  | Variety(type) | |
| --- | --- | --- | --- | --- | --- | --- | --- | --- | --- |
|  |  |  |  |  | *df* | *F* |  | *df* | *F* |
| *Ψ*_12_ | -1.86 ± 0.05^b^ | -1.21 ± 0.16^a^ | -1.78 ± 0.11^b^ |  | 2 | 13.82* |  | 27 | 3.86* |
| *Ψ*_50_ | -2.74 ± 0.03^b^ | -2.41 ± 0.13^a^ | -3.20 ± 0.07^c^ |  | 2 | 44.42* |  | 27 | 6.08* |
| *Ψ*_88_ | -3.63 ± 0.04^a^ | -3.62 ± 0.14^a^ | -4.62 ± 0.10^b^ |  | 2 | 59.06* |  | 27 | 4.81* |
| *S* | 66.92 ± 2.46^a^ | 43.90 ± 2.91^b^ | 36.53 ± 1.82^b^ |  | 2 | 10.53* |  | 27 | 2.15* |

**Supplementary Table S7. Difference in stem vulnerability to xylem embolism among *Vitis vinifera* subspecies (proles).** Summer mean values (± standard errors) of xylem pressure inducing 12 %, 50 %, and 88 % loss of hydraulic conductivity (*Ψ*_12_, *Ψ*_50,_ and *Ψ*_88_, respectively; in MPa), and of the slope of the vulnerability curve at the inflection point (*S*; in % MPa^-1^) in *Vitis vinifera* subsp. (proles) occidentalis, orientalis and pontica. Measurements were carried out on one-year-old plants. *n* = 77, 49, and 13 plants for *Vitis vinifera* subsp. (proles) occidentalis, orientalis, and pontica, respectively. *F* values refer to generalized linear models where type of varieties was treated as a fixed factor and variety nested within type of variety as a random factor. **P* < 0.05. Letters refer to Student-Newman-Keuls post hoc tests.

| Trait | Proles occidentalis | Proles orientalis | Proles  pontica |  | Subspecies | |  | Variety(subsp) | |
| --- | --- | --- | --- | --- | --- | --- | --- | --- | --- |
|  |  |  |  |  | *df* | *F* |  | *df* | *F* |
| *Ψ*_12_ | -1.94 ± 0.07^ab^ | -1.70 ± 0.06^a^ | -2.06 ± 0.12^b^ |  | 2 | 3.54* |  | 19 | 3.97* |
| *Ψ*_50_ | -2.85 ± 0.04^b^ | -2.59 ± 0.04^a^ | -2.72 ± 0.06^b^ |  | 2 | 7.43* |  | 19 | 5.10* |
| *Ψ*_88_ | -3.77 ± 0.05^b^ | -3.47 ± 0.05^a^ | -3.38 ± 0.03^a^ |  | 2 | 9.09* |  | 19 | 3.70* |
| *S* | 66.54 ± 3.57^b^ | 63.17 ± 3.40^b^ | 83.35 ±7.95^a^ |  | 2 | 2.88* |  | 19 | 2.21* |

**Supplementary Table S8. Difference in stem vulnerability to xylem embolism among *Vitis vinifera* geographical groups.** Summer mean values (± standard errors) of xylem pressure inducing 12 %, 50 %, and 88 % loss of hydraulic conductivity (*Ψ*_12_, *Ψ*_50,_ and *Ψ*_88_, respectively; in MPa), and of the slope of the vulnerability curve at the inflection point (*S*; in % MPa^-1^) in *Vitis vinifera* plants originating from Balkans (BALK), Eastern Mediterranean & Caucasus (EMCA), Iberian Peninsula (IBER) and Western & Central Europe (WCEUR). Measurements were carried out on one-year-old plants. *n* = 9, 17, 5, and 108 plants for BALK, EMCA, IBER, and WCEUR, respectively. *F* values refer to generalized linear models where type of varieties was treated as a fixed factor and variety nested within type of variety as a random factor. **P* < 0.05. Letters refer to Student-Newman-Keuls post hoc tests.

|  |  |  |  |  |  | Group | |  | Variety(group) | |
| --- | --- | --- | --- | --- | --- | --- | --- | --- | --- | --- |
| Trait | BALK | EMCA | IBER | WCEUR |  | *df* | *F* |  | *df* | *F* |
| *Ψ*_12_ | -1.59 ± 0.23^a^ | -1.69 ± 0.09^a^ | -1.70 ± 0.20^a^ | -1.92 ± 0.05^a^ |  | 3 | 1.25 |  | 18 | 4.32* |
| *Ψ*_50_ | -2.40 ± 0.16^a^ | -2.56 ± 0.06^a,b^ | -2.74 ± 0.07^b^ | -2.80 ± 0.03^b^ |  | 3 | 7.80* |  | 18 | 5.45* |
| *Ψ*_88_ | -3.20 ± 0.13^a^ | -3.43 ± 0.07^a,b^ | -3.79 ± 0.17^c^ | -3.68 ± 0.04^c,b^ |  | 3 | 8.21* |  | 18 | 4.27* |
| *S* | 67.31 ± 5.99^a^ | 60.35 ± 3.40^a^ | 52.56 ± 7.87^a^ | 68.59 ± 3.04^a^ |  | 3 | 59.00 |  | 18 | 2.50* |

**Supplementary Table S9. Difference in stem vulnerability to xylem embolism between *Vitis vinifera* berry skin colors.** Summer mean values (± standard errors) of xylem pressure inducing 12 %, 50 %, and 88 % loss of hydraulic conductivity (*Ψ*_12_, *Ψ*_50,_ and *Ψ*_88_, respectively; in MPa), and of the slope of the vulnerability curve at the inflection point (*S*; in % MPa^-1^) in *Vitis vinifera* black-berried and white-berried varieties. Measurements were carried out on one-year-old plants. *n* = 97, and 42 plants for *Vitis vinifera* black-berried and white-berried varieties, respectively. *F* values refer to generalized linear models where type of varieties was treated as a fixed factor and variety nested within type of variety as a random factor. **P* < 0.05. Letters refer to Student-Newman-Keuls post hoc tests.

| Trait | Black-berried varieties | White-berried varieties |  |  | Skin |  | Variety(skin) | |
| --- | --- | --- | --- | --- | --- | --- | --- | --- |
|  |  |  |  | *df* | *F* |  | *df* | *F* |
| *Ψ*_12_ | -1.91 ± 0.05^a^ | -1.76 ± 0.09^a^ |  | 1 | 1.12 |  | 20 | 4.18* |
| *Ψ*_50_ | -2.80 ± 0.03^b^ | -2.62 ± 0.05^a^ |  | 1 | 10.71* |  | 20 | 5.76* |
| *Ψ*_88_ | -3.69 ± 0.04^b^ | -3.47 ± 0.06^a^ |  | 1 | 11.94* |  | 20 | 4.42* |
| *S* | 66.52 ± 3.00^a^ | 67.84 ± 4.30^a^ |  | 1 | 0.11 |  | 20 | 2.39* |

**Supplementary Table S10.** **Xylem anatomical traits.** Mean values (± standard errors) of xylem area (*A*_xyl_, in mm²), equivalent circle diameter (*D*, in µm), weighted hydraulic diameter (*D*_H_, in µm), theoretical specific hydraulic conductivity *(k*_th_, in kg s^-1^ m^-1^ MPa^-1^) and xylem vessel density (*V*_D_, number of vessels per mm^2^) in 19 grapevine varieties. Observations were carried out on one-year-old plants for which vulnerability to xylem embolism was measured during the summer 2018. Numbers in brackets refer to sample sizes. Letters refer to Student-Newman-Keuls post hoc tests from one-way analyses of variance. Different letters mean statistically significant differences.

| Variety | *A*_xyl_ | *D* | *D*_H_ | *k*_th_ | *V*_D_ |
| --- | --- | --- | --- | --- | --- |
| *Vitis vinifera* |  |  |  |  |  |
| Cabernet franc (2) | 25.57 ± 0.61^ab^ | 37.48 ± 1.59^b^ | 132.84 ± 0.02^a-d^ | 57.25 ± 3.50^cd^ | 88.72 ± 8.71^a^ |
| Cabernet Sauvignon (3) | 23.45 ± 1.93^ab^ | 45.39 ± 4.62^ab^ | 148.77 ± 2.20^abc^ | 80.23 ± 7.03^cd^ | 68.96 ± 11.12^a^ |
| Chardonnay (3) | 18.54 ± 0.68^ab^ | 37.23 ± 2.86^b^ | 131.49 ± 6.30^a-d^ | 75.70 ± 16.74^cd^ | 90.82 ± 6.35^a^ |
| Cinsaut (2) | 23.94 ± 3.93^ab^ | 59.30 ± 1.15^ab^ | 140.19 ± 6.48^a-d^ | 124.61 ± 3.74^abc^ | 58.35 ± 4.30^a^ |
| Clairette (3) | 39.92 ±8.80^a^ | 65.54 ± 3.13^a^ | 165.52 ± 7.13^a^ | 160.85 ± 15.91^ab^ | 43.97 ± 3.35^a^ |
| Grenache (2) | 33.54 ± 5.10^ab^ | 50.00 ± 7.69^ab^ | 149.39 ± 9.12^abc^ | 131.72 ± 3.18^abc^ | 94.82 ± 40.00^a^ |
| Merlot (4) | 19.82 ± 1.69^ab^ | 47.05 ± 6.17^ab^ | 133.75 ± 8.03^a-d^ | 114.37 ± 6.43^abc^ | 103.34 ± 29.02^a^ |
| Pinot noir (2) | 15.76 ± 0.67^ab^ | 40.30 ± 1.49^ab^ | 126.85 ± 5.83^bcd^ | 72.63 ± 10.41^cd^ | 91.92 ± 11.07^a^ |
| Saperavi (3) | 14.25 ± 1.53^b^ | 48.49 ± 0.22^ab^ | 124.47 ± 4.50^cd^ | 97.37 ± 11.38^a-d^ | 98.29 ± 17.58^a^ |
| Sauvignon blanc (2) | 25.11 ± 1.76^ab^ | 45.25 ± 0.64^ab^ | 166.46 ± 7.90^a^ | 123.97 ± 15.75^abc^ | 76.50 ± 5.86^a^ |
| Sultanine (4) | 32.14 ± 8.58^ab^ | 55.48 ± 5.89^ab^ | 143.64 ± 9.75^abc^ | 123.22 ± 18.22^abc^ | 76.84 ± 12.46^a^ |
| Syrah (3) | 21.11 ± 2.67^ab^ | 41.00 ± 2.87^ab^ | 118.25 ± 2.33^cd^ | 60.91 ± 1.40^cd^ | 99.37 ± 6.13^a^ |
| Tempranillo (3) | 18.23 ± 1.01^ab^ | 42.31 ± 1.26^ab^ | 131.74 ± 8.51^a-d^ | 122.59 ± 32.65^abc^ | 132.27 ± 32.90^a^ |
| Tinta pinheira (2) | 21.35 ± 0.23^ab^ | 41.68 ± 3.13^ab^ | 124.91 ± 5.69^cd^ | 79.63 ± 13.55^cd^ | 95.77 ± 11.89^a^ |
| Ugni blanc (3) | 20.69 ± 1.44^ab^ | 52.09 ± 4.08^ab^ | 162.61 ± 5.47^ab^ | 166.56 ± 11.10^a^ | 75.10 ± 8.65^a^ |
| *Hybrid varieties* |  |  |  |  |  |
| Artaban (2) | 21.46 ± 3.50^ab^ | 36.93 ± 3.00^b^ | 105.83 ± 3.73^d^ | 33.02 ± 8.13^d^ | 82.06 ± 0.63^a^ |
| Floreal (2) | 33.03 ± 2.94^ab^ | 40.17 ± 4.22^ab^ | 143.20 ± 0.24^abc^ | 96.92 ± 5.13^a-d^ | 86.83 ± 9.57^a^ |
| Vidoc (2) | 18.78 ± 1.42^ab^ | 45.80 ± 12.52^ab^ | 130.74 ± 0.41^a-d^ | 87.21 ± 1.51^bcd^ | 85.92 ± 29.52^a^ |
| Voltis (3) | 32.92 ± 2.93^ab^ | 55.39 ± 8.01^ab^ | 134.34 ± 4.21^a-d^ | 93.22 ± 12.47^a-d^ | 59.56 ± 10.24^a^ |

**Supplementary Table S11. Difference in xylem anatomical traits among types of grapevine varieties.** Mean values (± standard errors) of xylem area (*A*_xyl_, in mm²), equivalent circle diameter (*D*, in µm), weighted hydraulic diameter (*D*_H_, in µm), theoretical specific hydraulic conductivity (*k*_th_, in kg s^-1^ m^-1^ MPa^-1^), and xylem vessel density (*V*_D_, number of vessels per mm^2^) in *Vitis vinifera* and hybrid varieties. Measurements were carried out on one-year-old plants. *n* = 41 and 9 plants for *Vitis vinifera* and hybrid varieties, respectively. *F* values refer to generalized linear models where type of varieties was treated as a fixed factor and variety nested within type of variety as a random factor. **P* < 0.05. Letters refer to Student-Newman-Keuls post hoc tests.

| Trait | *Vitis vinifera* | Hybrids |  |  | Type |  | Variety(type) | |
| --- | --- | --- | --- | --- | --- | --- | --- | --- |
|  |  |  |  | *df* | *F* |  | *df* | *F* |
| *A*_xyl_ | 23.58 ± 1.47^a^ | 27.25 ± 2.56^a^ |  | 1 | 1.18 |  | 17 | 2.55* |
| *D* | 47.53 ± 1.57^a^ | 45.77 ± 4.16^a^ |  | 1 | 0.63 |  | 17 | 2.63* |
| *D*_H_ | 139.99 ± 2.79^a^ | 129.17 ± 4.86^b^ |  | 1 | 7.53* |  | 17 | 4.93* |
| *k*_th_ | 107.87 ± 6.12^a^ | 79.33 ± 9.67^b^ |  | 1 | 9.87* |  | 17 | 5.01* |
| *V*_D_ | 86.81 ± 5.16^a^ | 76.48 ± 7.33^a^ |  | 1 | 0.46 |  | 17 | 1.21 |

**Supplementary Table S12. Relationships between hydraulic (*Ψ*_12,_ *Ψ*_50_ and *Ψ*_88_) and xylem anatomical traits (*A*_xyl_, *D*, *D*_H_, *k*_th_ and *V*_D_) among grapevine varieties.** *Ψ*_12_, *Ψ*_50_ and *Ψ*_88_ (in MPa) refer to xylem pressure inducing 12 %, 50 %, and 88 % loss of hydraulic conductivity, *A*_xyl_ to the xylem area (in mm²), *D* to the equivalent circle diameter (in µm), *D*_H_ to the weighted hydraulic diameter (in µm), *k*_th_ to the theoretical specific hydraulic conductivity (in kg s^-1^ m^-1^ MPa^-1^), and *V*_D_ to xylem vessel density (number of vessels per mm^2^). Measurements were carried out on one-year-old plants. *F* values refer to generalized linear models where variety was treated as a random factor.

|  |  |  | *Ψ*_12_ |  |  |  | *Ψ*_50_ |  |  |  | *Ψ*_88_ |
| --- | --- | --- | --- | --- | --- | --- | --- | --- | --- | --- | --- |
|  | *df* | *F* | *P* |  | *df* | *F* | *P* |  | *df* | *F* | *P* |
| *A*_xyl_ | 1, 28 | 0.85 | 0.3658 |  | 1, 28 | 0.10 | 0.7595 |  | 1, 28 | 0.11 | 0.7479 |
| *D* | 1, 28 | 0.08 | 0.7859 |  | 1, 28 | 0.17 | 0.6867 |  | 1, 28 | 2.70 | 0.1115 |
| *D*_H_ | 1, 28 | 0.95 | 0.3378 |  | 1, 28 | 1.87 | 0.1819 |  | 1, 28 | 0.99 | 0.3278 |
| *k*_th_ | 1, 28 | 0.65 | 0.4270 |  | 1, 28 | 3.36 | 0.0774 |  | 1, 28 | 4.07 | 0.0533 |
| *V*_D_ | 1, 28 | 0.00 | 0.9806 |  | 1, 28 | 0.00 | 0.9581 |  | 1, 28 | 0.30 | 0.5877 |


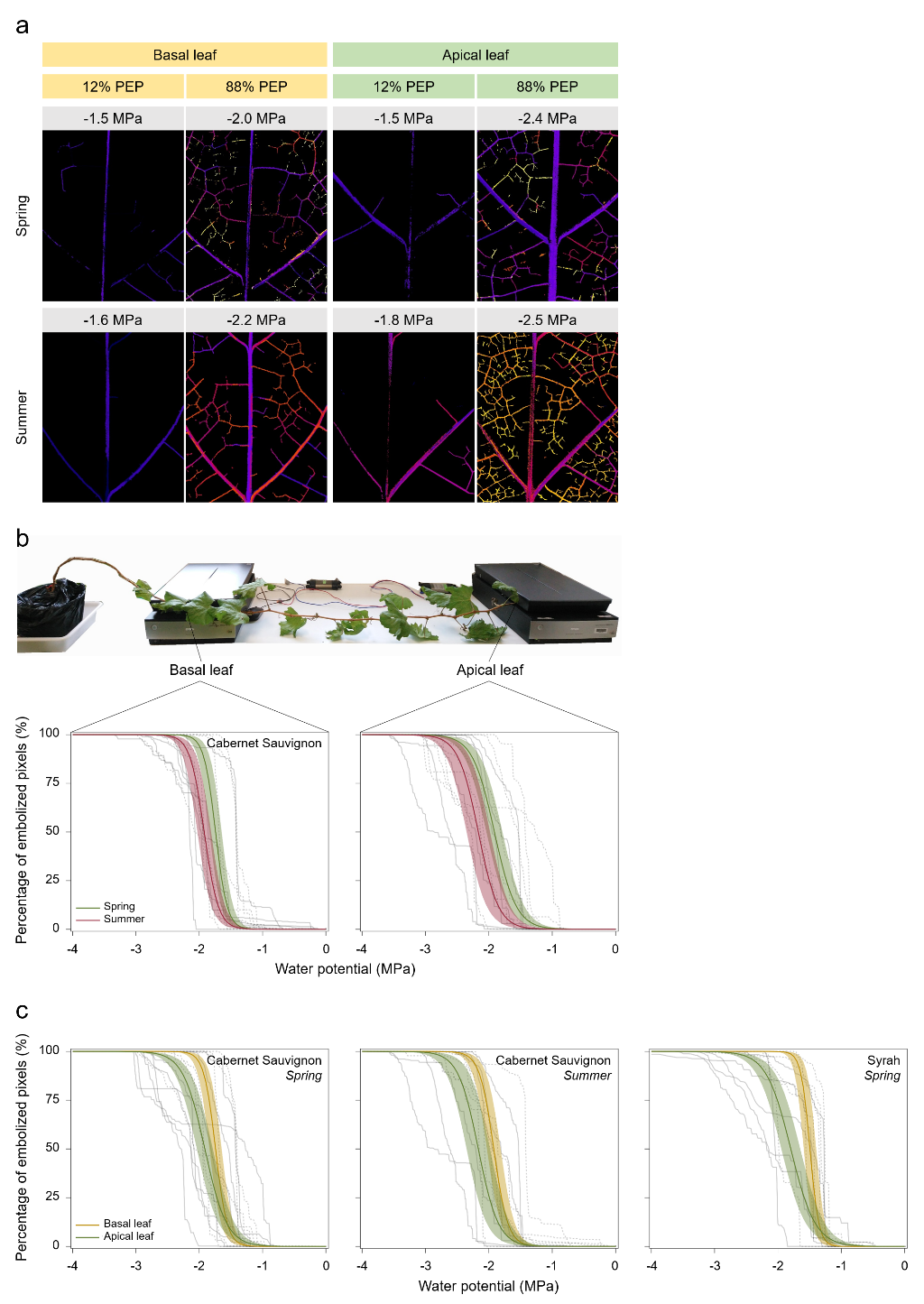


Supplementary Fig. S1. Variability in leaf vulnerability to xylem embolism. (a) Digital representation of embolism accumulation in basal and apical leaves of Cabernet Sauvignon plants in spring and summer. (b) Mean spring and summer optical vulnerability curves (VCs), expressed as percentage of embolized pixels (PEP, %), for basal and apical leaves of Cabernet Sauvignon plants. Shaded bands represent standard errors, while dashed and solid grey curves represent spring and summer raw data, respectively. (c) Mean optical vulnerability curves for basal and apical leaves of Cabernet Sauvignon and Syrah plants in spring and summer. Shaded bands represent standard errors, while dashed and solid grey curves represent basal and apical leaf raw data, respectively. All measurements were conducted on one-year-old plants, with 1 basal and 1 apical leaf measured per plant, using the optical vulnerability technique. See Supplementary Table S4 for sample sizes, means ± SE and results of statistical tests.


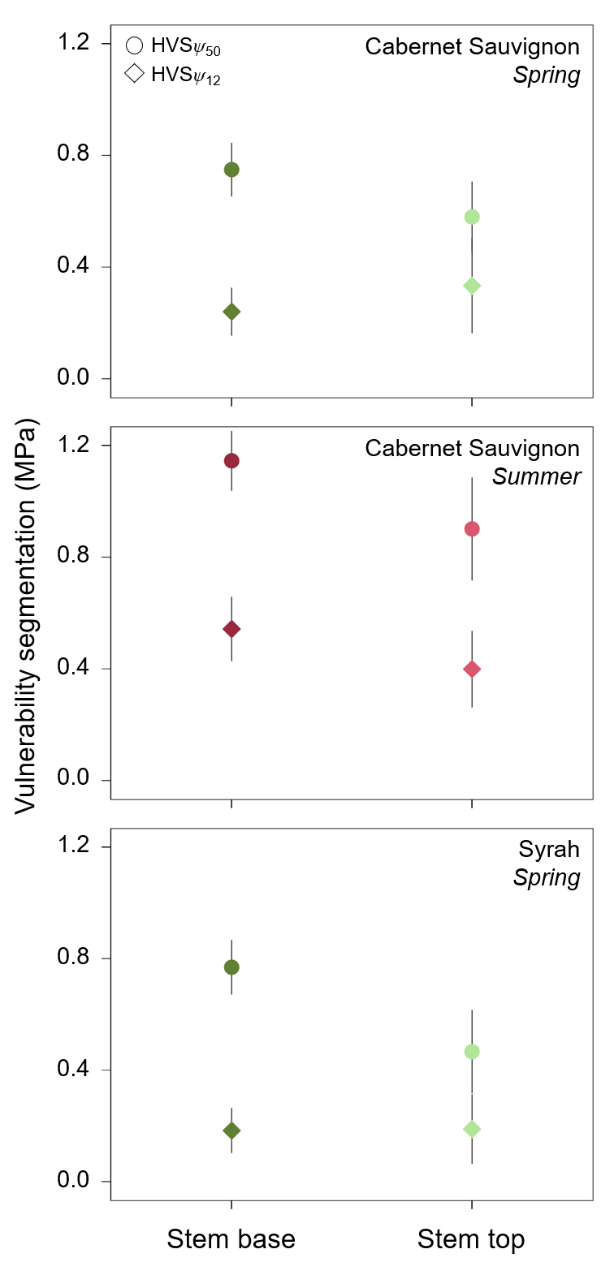


Supplementary Fig. S2. Hydraulic vulnerability segmentation in Cabernet Sauvignon and Syrah. The differences in *Ψ*_12_ and *Ψ*_50_ between leaves and stems (expressed as HVS_Ψ12_ and HVS_Ψ50_, respectively) were calculated in Cabernet Sauvignon and Syrah in spring and summer and for both basal and apical leaves of each plant measured with the OV technique, using the mean stem *Ψ*_12_ and *Ψ*_50_ per variety and season from the flow-centrifuge technique measurements. See Supplementary Table S4 for sample sizes, means ± SE and results of statistical tests.

**
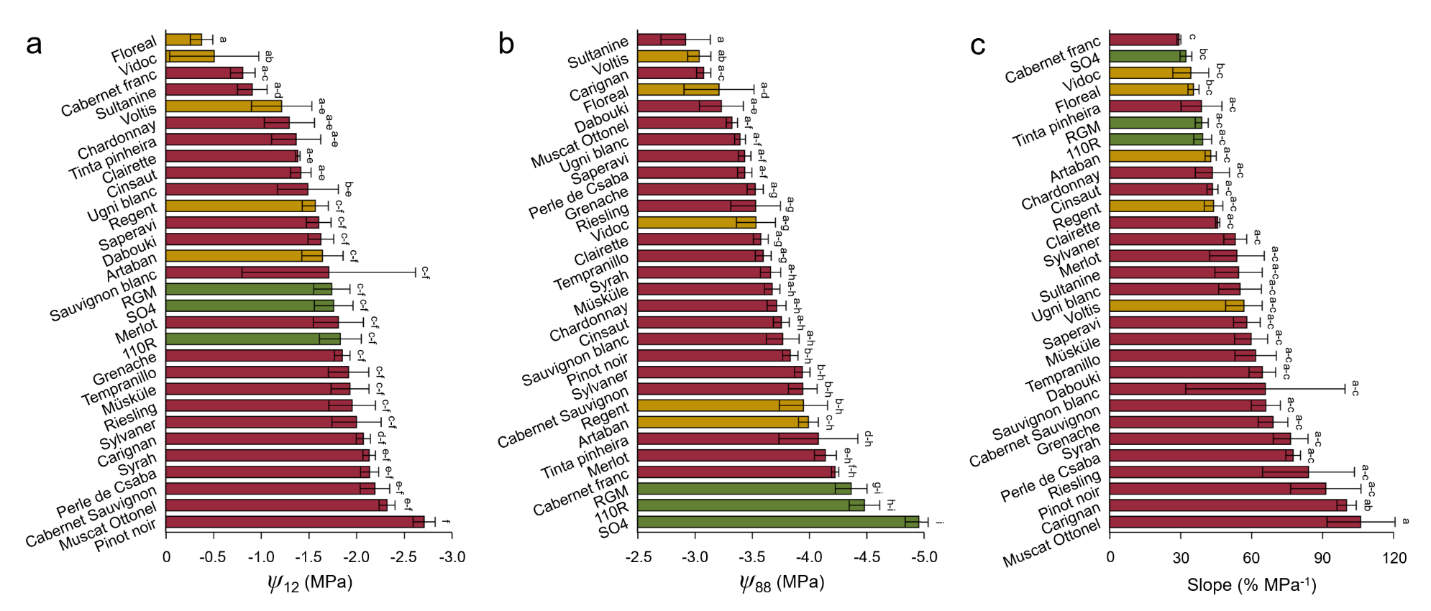
**

Supplementary Fig. S3. Variability in stem vulnerability to xylem embolism among grapevine varieties. Range of (a) *Ψ*_12_ (%), (b) *Ψ*_88_ (%), and (c) slope of vulnerability curves
(% MPa^-1^) for the 30 grapevines screened. All measurements were carried out in summer. Bars and errors represent means ± standard errors. Letters refer to the results of post-hoc tests. Different letters indicate significant differences among varieties. See Supplementary Table S5 for means ± SE.


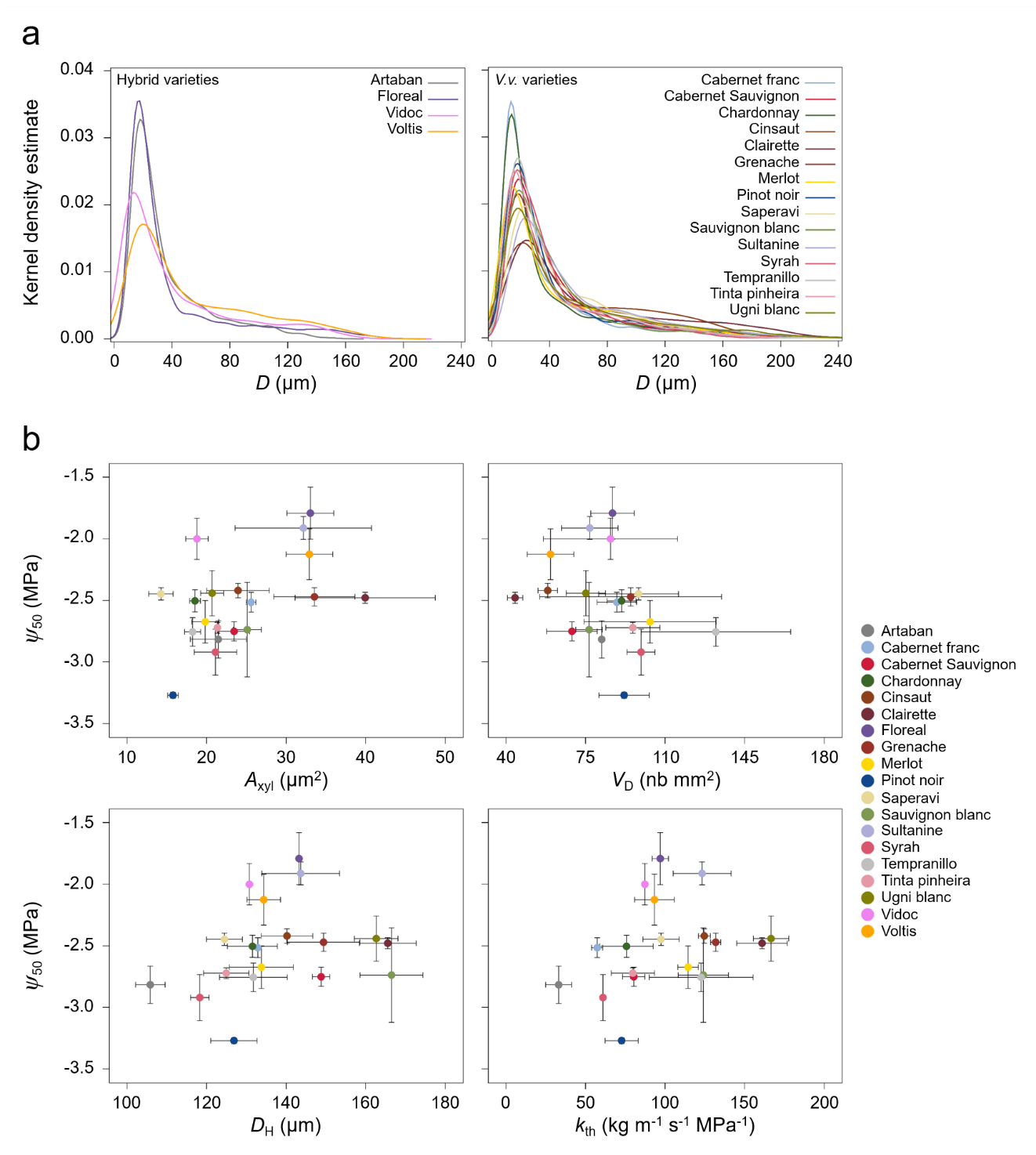


Supplementary Fig. S4. Xylem anatomy comparison among 19 grapevine varieties. (a) Box plots representing for each variety the proportion of xylem vessels per class of vessel diameter. (b) Relationships between hydraulic (*Ψ*_50_) and xylem anatomical traits (*A*_xyl_, *V*_D_, *D*_H_ and *k*_th_), where *Ψ*_50_ (in MPa) refers to xylem pressure inducing 50 % xylem embolism, *A*_xyl_ (in m²) to xylem area, *V*_D_ (number of vessels per mm^2^) to xylem vessel density, *D*_H_ (in µm) to weighted hydraulic diameter, and *k*_th_ (in kg s^-1^ m^-1^ MPa^-1^) to the theoretical specific hydraulic conductivity.


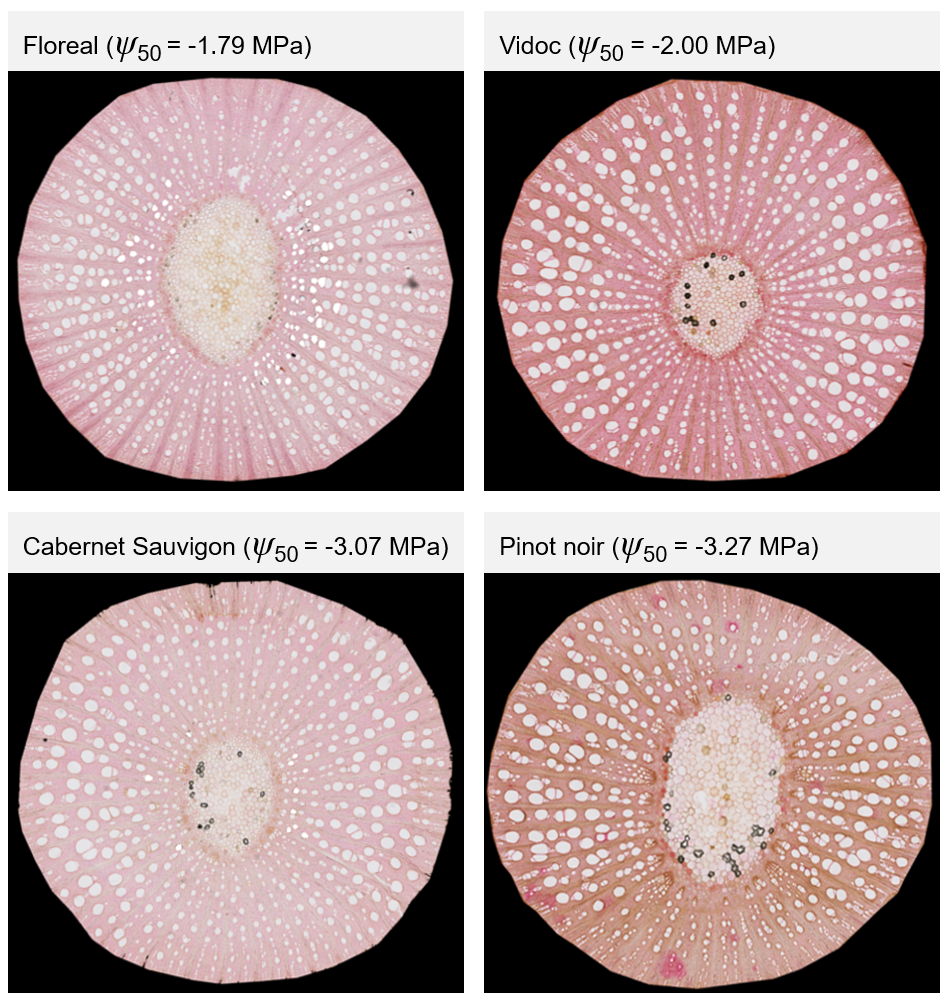


**Supplementary Fig. S5.** **Xylem anatomy.** Cross-sections of one-year-old stems of hybrid (Floreal and Vidoc) and *Vitis vinifera* (Cabernet Sauvignon and Pinot noir) varieties (scale bar = 5 mm). Xylem anatomy observations were made on stems that were previously used to measure xylem embolism vulnerability with the flow-centrifugation technique.


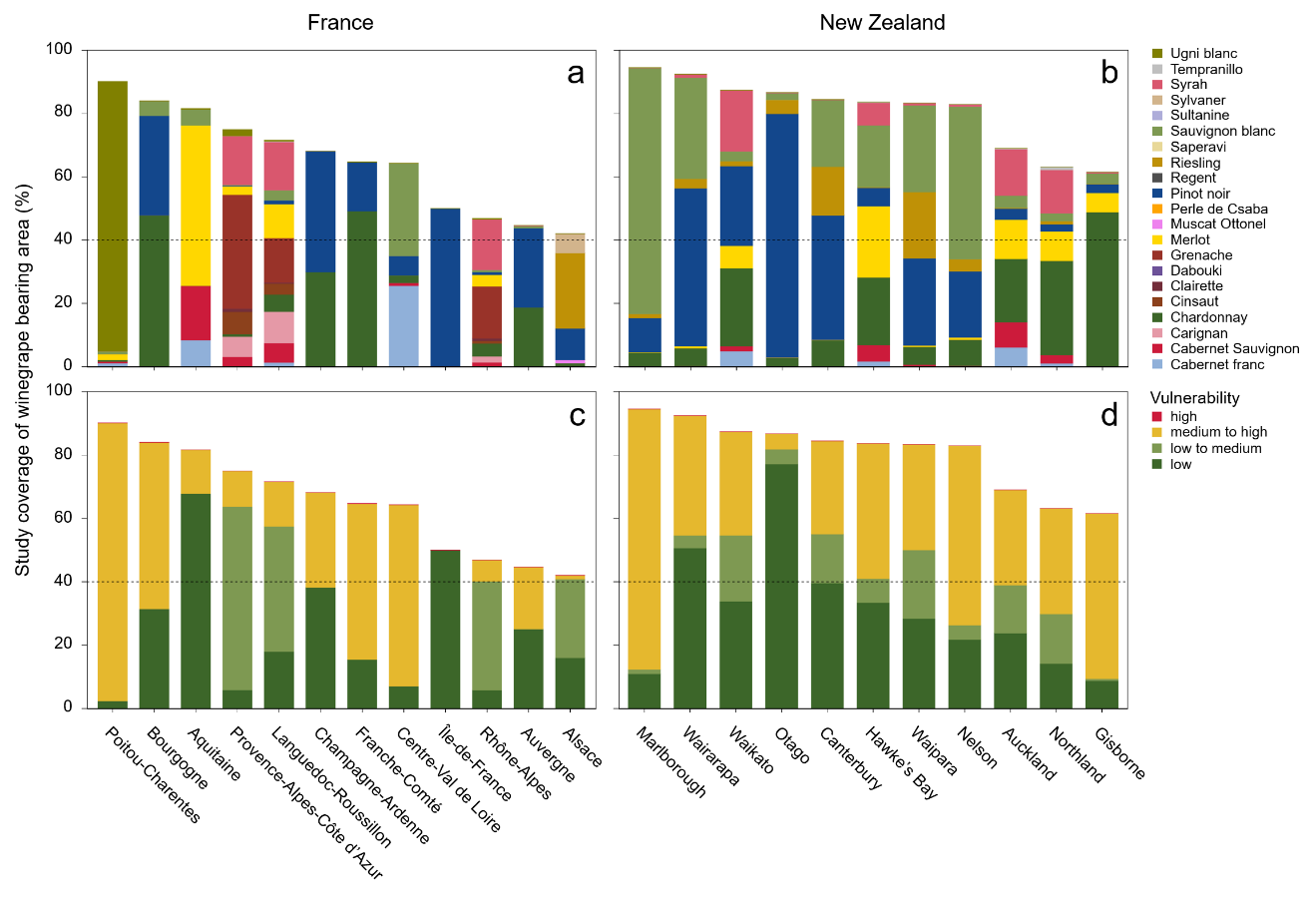


**Supplementary Fig. S6.** **Study coverage of regional winegrape bearing areas.** (a,b) Cumulative proportion of regional bearing area covered by the *Vitis vinifera* varieties included in this study, for winegrowing regions in France and New Zealand. (c,d) Proportion of regional bearing area covered by each cluster of vulnerability to xylem embolism (low, low-to-medium, medium-to-high, high), for winegrowing regions in (c) France and (d) New Zealand. The four clusters were previously identified from a hierarchical clustering analysis (see Supplementary Figure 2). Horizontal dotted lines indicate the 40 % regional coverage threshold that was used to represent the regional index of drought vulnerability. Note that the varietal coverage was compiled for all the 22 *Vitis vinifera* varieties studied but Müsküle, for which online data were not available.


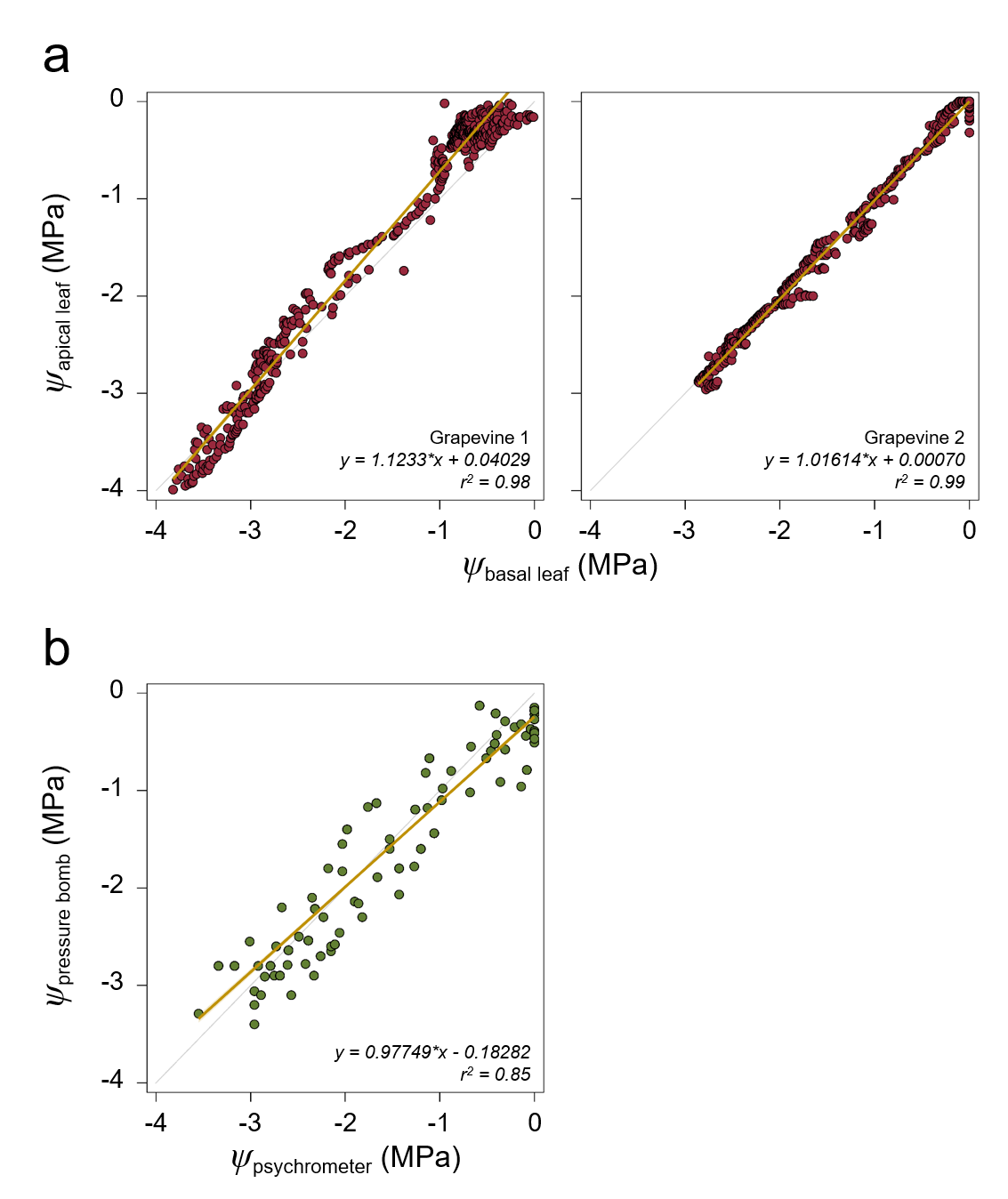


**Supplementary Fig. S7.** **Accuracy of water potential measurements when running the optical vulnerability technique.** (a) Relationship between water potentials recorded near the apical leaves (*Ψ*_apical leaf_) and those recorded near the basal leaves (*Ψ*_basal leaf_), using stem psychrometers. (b) Relationship between water potentials recorded simultaneously with stem psychrometers and Scholander pressure bomb. In all cases the regression line (solid yellow line) did not significantly differ from the 1:1 line (solid grey line; *P* < .05).
